# Recovery of the poisoned topoisomerase II for DNA religation: coordinated motion of the cleavage core revealed with the microsecond atomistic simulation

**DOI:** 10.1093/nar/gkv672

**Published:** 2015-07-06

**Authors:** Nan-Lan Huang, Jung-Hsin Lin

**Affiliations:** 1 Research Center for Applied Sciences, Academia Sinica, Nangang, Taipei 11529, Taiwan; 2 Institute of Biomedical Sciences, Academia Sinica, Nangang, Taipei 11529, Taiwan; 3 School of Pharmacy, National Taiwan University, Taipei 10050, Taiwan

## Abstract

Type II topoisomerases resolve topological problems of DNA double helices by passing one duplex through the reversible double-stranded break they generated on another duplex. Despite the wealth of information in the cleaving operation, molecular understanding of the enzymatic DNA ligation remains elusive. Topoisomerase poisons are widely used in anti-cancer and anti-bacterial therapy and have been employed to entrap the intermediates of topoisomerase IIβ with religatable DNA substrate. We removed drug molecules from the structure and conducted molecular dynamics simulations to investigate the enzyme-mediated DNA religation. The drug-unbound intermediate displayed transitions toward the resealing-compliant configuration: closing distance between the cleaved DNA termini, *B*-to-*A* transformation of the double helix, and restoration of the metal-binding motif. By mapping the contact configurations and the correlated motions between enzyme and DNA, we identified the indispensable role of the linker preceding winged helix domain (WHD) in coordinating the movements of TOPRIM, the nucleotide-binding motifs, and the bound DNA substrate during gate closure. We observed a nearly vectorial transition in the recovery of the enzyme and identified the previously uncharacterized roles of Asn508 and Arg677 in DNA rejoining. Our findings delineate the dynamic mechanism of the DNA religation conducted by type II topoisomerases.

## INTRODUCTION

DNA topoisomerases disentangle DNA double helices from the topological problems generated during replication, transcription and other critical biological processes. Type I topoisomerases are monomeric enzymes that alter the supercoiled form by nicking and closing single strand of the DNA. In contrast, type II topoisomerases are dimeric enzymes that catalyze ATP-dependent DNA strand passage through the transient double-stranded break they generated on another DNA segment (1–5). In the absence of either Mg^2+^ or ATP, topoisomerases will bind their substrate DNA and turn into a non-covalent complex (6,7). In the presence of Mg^2+^, however, the enzyme rapidly establishes a cleavage/religation equilibrium on the substrate DNA prior to the strand passage event (7,8). When the phosphodiester bonds of the substrate DNA are cleaved via the active site tyrosyl residues in the core domain of the enzyme (9), the noncovalent pre-cleavage complex turns into the covalent enzyme•DNA intermediate that is dubbed the ‘cleavage complex’ (10). This cleavage complex retains tyrosyl phosphate linkage to the newly generated 5′-termini of the broken DNA (10). Upon ATP binding, the enzyme transports an intact doubly helical strand through the transient break in the bound nucleotides and then establishes another cleavage/religation equilibrium of the bound DNA after the strand passage operation (7). Hydrolysis of the bound ATP molecules allows the enzyme to initiate a new catalytic cycle (11). The use of topoisomerase poisons can trap the covalent cleavage complex by impeding the rejoining of the broken DNA, either prior to or after the strand passage operation, given the drug molecules are present *in situ* at the instant of DNA cleavage (7,12). These topoisomerase poisons have been widely used in anti-cancer and anti-bacterial therapy.

The topological rearrangements of DNA duplex accomplished by type II topoisomerase involve the controlled association and dissociation of distinct dimerized interfaces that are termed the ‘gates’ (13). The IIA subgroup of these enzymes, including the eukaryotic topoisomerase II (Top2) and prokaryotic topoisomerase IV (Top4), possess three such interfaces, which are designated the N gate, the DNA gate, and the C gate, respectively. In the scheme of the ‘two-gate’ mechanism for strand passage (14–16), the intact strand enters through the N gate, which is controlled by reversible dimerization of the ATPase domains, passes over the prised-open DNA gate and exits through the C gate, the interface at the carboxyl side of this machinery. The DNA gate, situated in the interior of the holoenzyme, comprises the metal-binding TOpoisomerase/ PRIMase (TOPRIM) domain (17) and the nucleotide-binding winged-helix domain (WHD) that contains the catalytic tyrosine (18). The DNA gate and the tower domains, which support nucleotide binding, arrange in pairs and function as the cleavage core of the enzyme. Although the participation of ATP is required in strand passage, it was reported that the presence of Mg^2+^ alone could support occasional opening of the DNA gate (19,20), indicating the ability of the enzyme to generate double-stranded break of gate-DNA in the absence of ATP. The truncated forms of topoisomerase II which only retain the cleavage core and the C gate have detectable DNA cleaving activity *in vitro* (21–23). In addition, the equivalent forms of topoisomerase IV has also been demonstrated to reseal the cleaved DNA in a drug-eluted crystal structure (24).

Characterization of the topoisomerase-mediated cleavage/religation used to be difficult because of the reversible nature of transphosphoryl reaction and the transitory attribute of the cleavage complex. To tackle this problem, modified DNA substrates were tailored to dissect the cleaving and the rejoicing activities of topoisomerase II (25–27). In addition, the use of phosphorothiolate DNA substrates was introduced in structural determination of topoisomerase (28). These substrates are competent for cleavage by topoisomerase but resistant to the enzyme-mediated rejoining and are widely used in crystallographic characterization of topoisomerase I and topoisomerase II (29–32). The abundant structural information has facilitated molecular simulations to reveal the dynamics of topoisomerase I and their DNA substrates (33–46). On the other hand, although recently the chemical reaction mechanism of topoisomerase II-mediated DNA cleavage/religation were investigated with the use of quantum mechanical/molecular mechanical (QM/MM) calculations at the picosecond time scale (47,48), the underlying molecular dynamics of the enzyme•DNA intermediate, which may require long-time relaxation of the very large structure of the enzyme-DNA complex, have not been characterized in these studies. Despite the extensive investigations into the cleaving operations of topoisomerase II, molecular understanding of the enzyme-mediated DNA ligation remains elusive. The only structural information on DNA resealing of type II topoisomerases was provided from the crystals of streptococcus topoisomerase IV by eluting the drugs out of the cleavage core (24). Sequential conformation changes of gate-DNA observed in the drug-bound complex, the drug-free complex, and the complex of resealed DNA have led to a putative mechanism of the drug-causing arrest during the reaction cycle of type II topoisomerase. In the case of eukaryotic topoisomerase II, however, similar drug-elution approach did not generate a similar intermediate structure with the ‘resealed’ DNA (49). In general, the conformational transitions of type II topoisomerase during the enzymatic DNA religation remain unclear.

Vertebrates express two isoforms of topoisomerase II, the IIα and IIβ isozymes (50–53). While the IIα isozyme is mainly involved in cell proliferation and serves as the therapeutic target for clinically used anti-cancer drugs (54), the housekeeping IIβ isozyme is constitutively expressed in postmitotic cells and turns out to play a crucial role in neural development (55,56). Both isozymes were observed to form detectable amounts of the cleavage complex in the presence of topoisomerase poisoning drugs. This observation has been exploited to determine the crystal structures of several drug-stabilized enzyme•DNA intermediates of the IIβ isozyme with resealable DNA substrates (23,49). Molecular dynamics simulations using these structures have revealed the conformational changes in an important side chain rotamer of the enzyme, triggered by drug binding (57). In addition to the promising potential in expediting new drug development, the use of religatable DNA substrate in these structures should facilitate elucidating the molecular dynamics of the DNA religation mediated by topoisomerase II.

In the current study, we utilized the drug-stabilized structure, removed the drug molecules from the complex, and exploited molecular dynamics simulations to investigate the topoisomerase II-mediated DNA rejoining operation. We observed the broken ends of the gate-DNA approached a religation-compliant distance. The occurrence of such distance on one strand of the gate-DNA was not concerted with that on the complementary strand. In addition, the gate-DNA demonstrated *B*-to-*A* transformations, as commonly observed in the complexes with enzymes capable of cutting or sealing phosphodiester linkage, and the helical curvature adopted by the resealed DNA in complex with topoisomerase. By mapping the probability of direct contacts and correlated motions between the enzyme and the DNA, we identified the role of the helix-bundle linker preceding WHD in coordinating the movements of the cleavage core and the bound nucleotides during the closing operation of the DNA gate. Our study establishes the connection of previous experimental finding and the dynamic mechanism in the initial stage of the DNA religation by type II topoisomerases.

## MATERIALS AND METHODS

### Molecular models and molecular dynamics simulations

Crystal coordinates of the etoposide-stabilized (PDB code: 3QX3) and the eluted drug-free (PDB code: 4J3N) cleavage complexes of human topoisomerase IIβ were used. The protein fragment determined in these structures comprised residues 450–1206, corresponding to the cleavage core and the C gate. Atomic coordinates of missing residues in the crystal structures were assigned as described in the *Supplementary methods*. For the phosphotyrosyl bond on the cleaved 5′-terminus in the topoisomerase II complex, we employed the two-stage RESP (58) fitting procedure to obtain the atomic charges (Supplementary methods; Supplementary Figure S1), based on crystal coordinates of the covalently linked Tyr821 and the +1 nucleotide. In the model of the drug-bound complex, the atomic charges of the drug molecules were derived using the AM1-BCC charge model (59,60). Protonation of amino acids were carried out with PDB2PQR (61,62). Molecular dynamics simulations were conducted using the AMBER 14 package (63). The topology and parameter files were constructed with tleap, using the ff99SB force field for protein (64,65) and the parmbsc0 modifications for nucleic acids (66). Systems were solvated with explicit solvent molecules using the TIP3P water model (67) and neutralized to simulate an environment of 150 mM sodium chloride aqueous solution. Each system contains more than 171 101 atoms in sum. Simulations were carried out with the particle-mesh Ewald method for calculating the full electrostatic interactions of a periodic box in the macroscopic lattice of repeating images. After energy minimizations, heating and equilibration at 310 K and 1 bar, NVT simulations were conducted with the time step of 2 fs and with the SHAKE constraints (68) on the bonds not involving hydrogen. The main simulation was carried out for 1000 ns, and the coordinates were recorded every 1 ps, generating 10^6^ snapshots for nucleic acid conformation analyses. The recorded conformations were sampled every 10 ps, generating 10^5^ snapshots for analyses of protein conformation.

### Nucleic acid and protein conformations

Nucleic acid conformations were analyzed using Curves+ (69). Time-dependent and probability analyses on nucleic acid conformations of the simulation trajectories were carried out using the *canal* module. RMSD were calculated using the cpptraj module of the AMBER program suite. Molecular graphics were generated using PyMOL or Chimera.

### Correlation analyses

The 50–200 ns periods of the simulations on the drug-unbound complexes (UD1-UD4) were selected to compare with that of the drug-bound complex (BD; Table 1). Each trajectory was sampled at an interval of 100 ps for the following analyses. The reference structure of each trajectory was selected by clustering using the cpptraj module of AMBER 14. The fitting of an ensemble of conformations from each trajectory was carried out by structural alignment with respect to the Cα atoms of the regions with well-folded secondary structures in its reference structure. Subsequently, the covariance analysis with the *nofit* option was conducted using gromacs-4.6.3, and the adequacy of sampling in each trajectory was tested with Hess's cosine-contents analysis (70). The cosine contents of the first principal components are sufficiently small (Supplementary Table S1), indicative of adequate level of convergence in the selected periods of simulations. Correlation analyses were carried out according to Lange and Grubmüller (71) using the g_correlation module of gromacs-3.3.3 with the *nofit* and the *linear* option, because this module is only implemented in earlier versions of gromacs. The method is based on mutual information and gives rise to ‘generalized’ correlation coefficients in the range of zero and unity. The correlation coefficient assumes the value of 1 for perfectly correlated motions and vanishes for completely uncorrelated motions. Both perfectly anti-correlated motion and perfectly correlated motion that are collinear in space, which are two special cases of correlated motions, will also give rise to the generalized correlation coefficient of 1. The correlation analyses were conducted on the 1500 Cα atoms of the protein and the forty C5′ atoms of the DNA.

**Table 1.** Systems of molecular dynamics simulations and relevant observations

**System**	Drug-unbound			Drug-bound	Drug-free
	UD1	UD2–4	UD5^b^	BD	FD
Starting structure (PDBID)	3QX3	3QX3	3QX3	3QX3	4J3N
Ligands in simulation	0	0	0	2	0
Simulation length	1000 ns	200 ns	100 ns	200 ns	200 ns

**Time-dependent transitions:**					
O3′(-1) : P(+1) distance	Significantly different on two strands^a,b^			n.a.	Shortening in one strand
DNA transformation	*B*-to-*A*	*B*-to-*A*	n.a.	n.a.	*B*-to-*A*
DNA helical curvature	‘Closed-interface’	n.a.	n.a.	n.a.	n.a.
Inter-WHD transition	‘Closed-interface’	n.a.	n.a.	n.a.	n.a.
Inter-**I872** distance	Shortening	Shortening	n.a.	Un-shortening	n.a.

**Equilibrium parameters:**					
*in*-*trans* contacts of <**EDxD**–cleavage site>	Higher prob.	Higher prob.	n.a.	Lower prob.	n.a.
*in*-*trans* contacts of <**R503**–cleavage site>	Higher prob.	Higher prob.	n.a.	Diminished	n.a.
*in*-*cis* contacts between <**N508**–DNA backbone>	Persistent	Persistent	n.a.	Persistent	n.a.
Pairs of correlated protein–DNA motions^c^	246	75–431	n.a.	35	n.a.
Correlated motions between linker and intercalating motifs^d^	78	14–101	n.a.	0	n.a.

^a^*P* < 0.0001 for the strand with the smaller average being indistinguishable from the complementary strand in the same complex.

^b^The UD5 simulation did not reveal apparent shortening of the distance and therefore was not included in the subsequent analyses.

^c^Paired protein–DNA motions with correlation coefficients ≥ 0.70.

^d^Paired motions between the linker (P659–L681) and the intercalating (E870–P880) residues, with correlation coefficients ≥ 0.70.

### Protein–DNA interactions

The 50–200 ns periods of simulations UD1–UD4 were analyzed and compared with the BD simulation. Probability of direct contact between each pair of amino acid and nucleic acid was calculated as: $p_c = \frac{2}{3}f_{3.5} + \frac{1}{3}f_{5.0}$, where *f_d_* indicates normalized frequency of the pairing residues being located within *d* Å to each other. In each conformation, the odds with respect to an indicated nucleotide assumes the value of 1 for an amino acid located within the 3.5-Å distance bound, 1/3 for that in the outer shell between 3.5 to 5 Å, and otherwise zero. The occurrence of an amino acid in contact with the indicated nucleotide is determined for the ensemble of conformations in each simulation trajectory and divided by the number of analyzed conformations. The probability of enzyme•DNA contacts were mapped with respect to the two polypeptide chains and the two oligonucleotide strands, giving rise to four pairing combinations of the contact configuration in each simulation system.

### Statistical analysis

The 1000-ns simulation trajectory of UD1, the 200-ns simulation trajectories of UD2-UD4, and the 100-ns trajectory of UD5 were used in two-way analysis of the variance (ANOVA) with unbalanced design using MATLAB. In regard to the distributions of the O3′(-1)—P(+1) distances, the oligonucleotide strand with the smaller average distance at issue from each trajectory was assigned to one group, and the larger to the other group of the first factor (Supplementary Table S2). The index of trajectory was used as the second factor. A factor is considered to have significant influence if the corresponding *p*-value is smaller than 0.001.

## RESULTS AND DISCUSSION

### Topoisomerase II directs the cleaved ends to a distance compliant for DNA religation

To delineate the underlying molecular dynamics of the topoisomerase II-mediated DNA religation, we used the structure of etoposide-stabilized cleavage complex, removed the drug molecules from the complex, and conducted molecular dynamics simulations starting from this conformation. We first set the double-stranded DNA apart from the enzyme and carried out molecular dynamics simulations of the ‘released’ double helix to test the validity of the newly derived charges (Supplementary methods). The double helix rapidly ‘relaxed’ from the curved, crystal conformation into *B*-DNA with a linear helical axis (Supplementary Figure S2), in concord with previous observations from simulations of solvated DNA molecules (72–75). The distances between O3′(−1) and P(+1) of the cleaved-apart nucleotide steps fluctuated drastically and frequently approached a lower bound of 3 Å (Supplementary Figure S1).

A pertinent process of the DNA religation by topoisomerase II has been observed in the cleavage complex of its bacterial homolog, the topoisomerase IV. Laponogov *et al*. exploited the slow resealing of DNA by topoisomerase IV cleavage complex, which was imposed by an antibacterial drug, to capture the stabilized structure (24). Subsequent elution of the drug molecules gave rise to a drug-free cleavage complex with the cleaved DNA, in which the O3′(−1) was situated 3.41 Å away from the P(+1). Remarkably, further incubation using another aliquot of the drug-free crystal with MgCl_2_ solution generated a complex with the DNA resealed. Structural basis for the sequential cleaving and rejoining steps of gate-DNA may implicate the residence of the cleavage complex with such O3′(−1)—P(+1) distance in a tentative local minimum of the energy landscape along the reaction pathway. It has also been observed in the precatalytic complex of DNA polymerase that O3′ of the primer terminus was situated within 3.4 Å from α-phosphorus of the incoming nucleoside triphosphate to be integrated (76). In addition, nucleophilic attacks between enzymes and substrate nucleotides have been successfully simulated starting from conformations with the target O—P distance of 3.08–3.5 Å, demonstrated in the QM/MM studies of DNA polymerase IV and RNase A (77,78). Accordingly, we selected 3 Å as the reference value in evaluating the O3′(-1)—P(+1) distances of the cleaved gate-DNA in the topoisomerase II complex.

**Figure 1.**
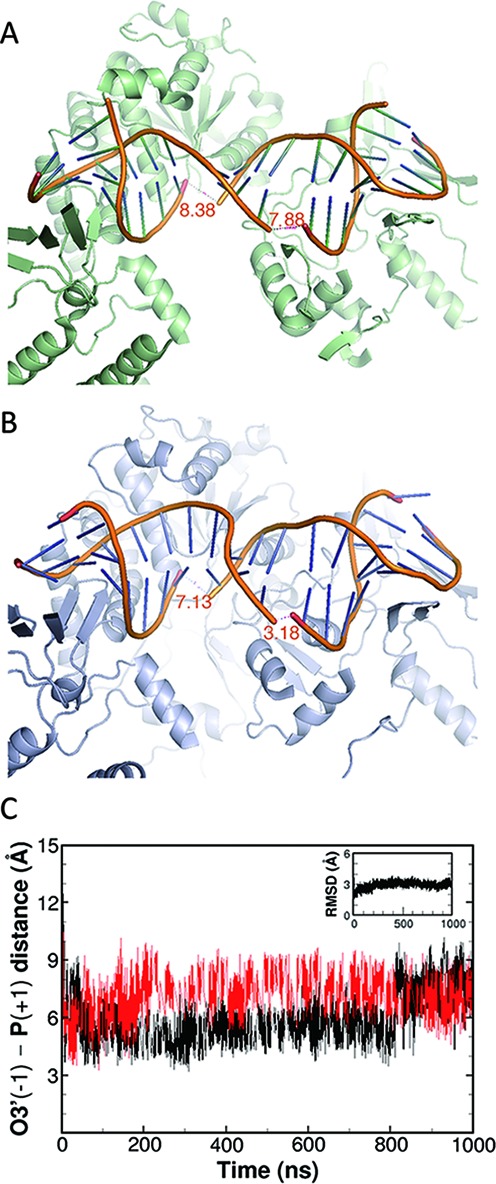
The cleaved DNA termini approach a rejoining-compliant distance in the drug-unbound (UD) cleavage complex of topoisomerase II. (**A**) crystal structure of the drug-bound complex (3QX3; drug not shown). (**B**) representative snapshot from simulation UD1. (**C**) O3′-P distances of the cleaved-apart nucleotides on the two strands along the 1-μs simulation of UD1. RMSD of the phosphodiester backbone is shown in the inset.

In contrast to the unbound double helix, the gate-DNA in complex with topoisomerase II was stabilized in a curved conformation (Figure 1A and B). To our surprise, even staying bound in the cleavage complex, the gaps between the cleaved termini descended toward the 3-Å baseline very quickly (Figure 1C). With the sampling frequency of 1 ps^−1^ during the microsecond simulation, we obtained the conformation with this distance of 3.18 Å (Figure 1B), comparable to the models in recent works using QM/MM geometry optimization to address the mechanism of this chemical reaction (47,48) (discussion in supplementary information; Supplementary Figure S3). To confirm this DNA religation process was indeed maneuvered by the enzyme, we carried out four additional independent simulations of the same commencing structure. In three out of the four repeating simulations, we also observed the rejoining-compliant O3′—P distance at times, indicating the capability of topoisomerase II to bring the cleaved ends to the resealing-compliant distance (Supplementary Figure S4, UD2-UD4). In the last repeating simulation, we observed the atom pairs departing from its initial distance of 8 Å and ascending to 14 Å (Supplementary Figure S4, UD5). The elongating gap in this simulation might implicate the opening of the entire DNA gate in the complex, which is beyond the scope of the current work. An interesting finding from comparison of these simulations is that we could hardly observe synchronized ligation of the cleaved ends on both strands of the gate-DNA. This intuitive observation was verified by the significant difference in the two strands in the simulations of the drug-unbound complex (*p* < 0.0001; Supplementary Table S2). Bromberg *et al*. developed a cleavage-independent DNA ligation assay for human topoisomerase II and found the enzyme tends to ligate the two scissile bonds in a non-concerted fashion (25,26). Because the nucleic acid sequence is palindromic in the structure used in the current study, it is particularly suitable for comparing such changes on the two strands. In addition to the simulations starting from the drug-stabilized crystal structure, we conducted another distinct simulation using an eluted *drug-free crystal* of topoisomerase IIβ (FD; Table 1 and Scheme S1) and observed one of the O3′(−1)—P(+1) distances moved toward the resealing-compliant value (Supplementary Figure S5). Our observations are consistent with experimental results on the DNA religation mediated by topoisomerase II.

### Predisposition of gate-DNA to the topoisomerase II-mediated religation

A remarkable attribute generally used in describing DNA is the tertiary arrangement of the double helix. Most crystallographic examples of complete *B*-to-*A* transformations occurred in complexes of DNA with enzymes that perform cutting or sealing operations at the O3′—P phosphodiester linkage. This transformation is proposed to expose the 3′-oxygen atoms, which was ordinarily buried within the backbone, for enzymatic attack (79). To gain a panoramic vision on the molecular dynamics in addition to a single distance criterion, we examined the nucleic acid conformations in the cleavage complex of topoisomerase II.

#### Slow Transition of the cleaved gate-DNA to A-form in the cleavage complex

**Figure 2.**
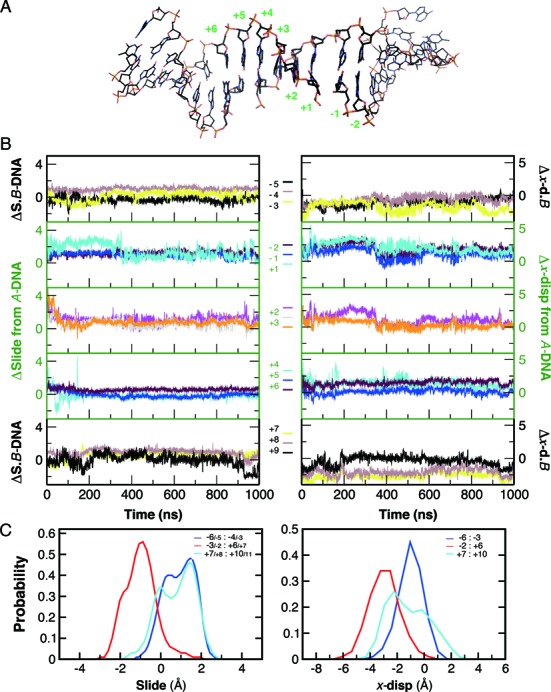
Transition of the gate-DNA to *A*-form in simulation UD1. (**A**) representative snapshot with −2 to +6 base pairs shown in thicker sticks. (**B**) Temporal transitions of Slide and *x*-displacement at individual base-pair/step. Difference in each parameter from that of the typical *A*-DNA was calculated for the +2 to +6 compartment (green frame), and difference from the value of typical *B*-DNA was calculated for the two flanking regions (black frame). The moving average of each parameter was calculated with a window size of 200 from 10^6^ snapshots. C, distributions of Slide and *x*-displacement of the gate-DNA in the 1-μs simulation. The clear partition in these distributions indicated stabilization of the −2 to +6 bp in *A-*form.

The discriminating power of the Slide and *x*-displacement (*x*-disp) between *A*-DNA and *B*-DNA have been pointed out in a comprehensive survey of nucleic acid structures (80). In the following context, we will simply refer to the residue index of the first strand, according to the assigned order in the crystal coordinates, to describe the analyzed loci of the conformation parameters. In the crystal structure of the drug-stabilized cleavage complex, the nucleotides settled on a global conformation of *B*-form (23) (Supplementary Figure S2). In the initial 200-ns of the main simulation, we observed an apparent decrease in Slide between the +3/+4 step and in *x*-disp of the +3 base-pair (Figure 2B). Subsequently, the −2 to +2 base-pairs displayed gradual lowering of *x*-disp, and the −1/+1 step also demonstrated a transition toward the typical Slide in *A*-DNA. With this trajectory conducted for up to 600-ns, distribution of both Slide and *x*-disp demonstrated a clear partition of the 20-bp DNA to a *B*-*A*-*B* configuration (not shown). The middle compartment (−3/−2 to +6/+7), which encloses the two scissile bonds, appeared to be stabilized in *A*-form during the entire 1-μs simulation (Figure 2C). Concordantly, we observed similar trends in Slide and *x*-disp during the 200-ns simulation of the drug-free complex (FD; Supplementary Figure S6 left). We also investigated the DNA transformations in the additional simulations of the drug-unbound complex. In contrast to the slow transition of the enclosed base pairs, the distributions of slide and *x*-disp in this period of simulations revealed an early settling of the flanking (−3/−2 and +6/+7) base pairs in *A*-form (Supplementary Figure S6, UD1–4 and FD).

The *B*-to-*A* transitions we observed in the middle compartment of the gate-DNA echo the crystallographic characterization of several type II topoisomerases (22,24,81). In the cleavage complex of streptococcus topoisomerase IV, this compartment (−2 to +6) displayed *A*-form arrangement in the drug-free complex but demonstrated the *B*-form in the drug-stabilized complex (24). Therefore, it was suggested that drug binding in the cleavage sites favored the ‘relaxation’ of the nucleotides into *B*-DNA. Furthermore, in the crystal of the human topoisomerase IIα cleavage core, the oligonucleotides that annealed to generate a 13-bp duplex with a complementary four-base, 5′-overhang was utilized as the substrate (82). As bound to the dimeric topoisomerase IIα, with one duplex to each enzyme protomer, the complementary overhangs rendered the nucleotides imitating a doubly nicked, 30-bp DNA substrate in the noncovalent complex. The nick on each strand in this IIα cleavage complex was not large enough to accommodate drug molecules; in contrast, the ‘slots’ in the *drug-bound complex of topoisomerase IIβ* appear to be expanded, owing to the changes in the global conformation of the dimeric enzyme. It was proposed that in accommodating drug molecules, the TOPRIM domain of the IIβ complex was rotated to drag the DNA gate open, and this movement decreases buried surface area by about 2000 Å^2^ in this compartment (82). On the contrary, since we removed the drug molecules from the complex, we would expect an increase in the buried surface area, which in turn should lead to a transition from *B*-DNA to A-form. In brief, our observation on the dynamic, conformational transition of the gate-DNA provides additional supports to previous findings. The enzyme-mediated remodeling of DNA to *A*-form may also provide a mechanism for smoothly bending the double helix and for accessing the minor groove edges of individual base-pairs (79).

#### Transition of helical curvature toward the ‘closed-interface’ configuration

**Figure 3.**
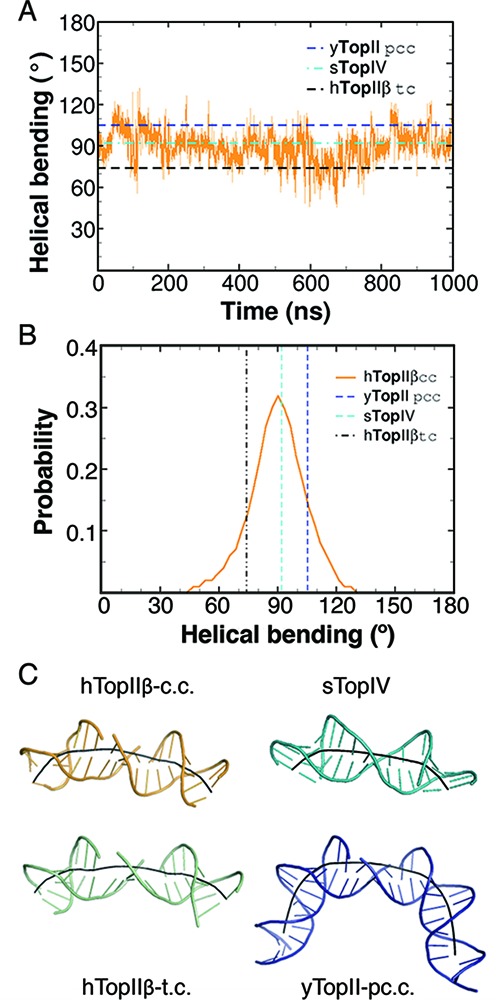
Helical curvatures of gate-DNA in simulation UD1 and the reference crystal structures. The internal 18-bp were used in curvilinear fitting. (A) helical bending along the 1-μs simulation. The moving average was calculated with a window size of 200 from 10^6^ snapshots. (B) helical bending revealed a shift toward those in the crystal structures with the closed ‘DNA gate’ of topoisomerases. (C) conformations of the gate-DNA in the representative snapshot from simulation UD1 (hTopIIβ-cc), the crystal of drug-bound topoisomerase II (hTopIIβ-tc; PDB code 3QX3), the pre-cleavage complex of yeast topoisomerase II (yTopII-pcc; PDB code 2RGR), and topoisomerase IV with the ‘resealed’ DNA (sTopIV; PDB code 3KSB). Helical axes were generated using Curves+.

The first crystallographic view on the DNA binding of topoisomerase II cleavage core distinguished the contribution of the conserved Ile residues (Ile833 in yeast Top2) to DNA *bending* by almost up to 150º (22). This enzyme-mediated bending of the gate-DNA was suggested to be a prerequisite to the cleavage reaction (83). We examined the extent of DNA bending in the 1-μs trajectory of topoisomerase II cleavage complex using the distinctive curvilinear analyses provided by the Curve+ program (69). To compare with the available crystal structures, we also investigated DNA bending in the pre-cleavage complex of yeast topoisomerase II, the drug-stabilized cleavage complex of human topoisomerase II, and the streptococcus topoisomerase IV with ‘resealed’ DNA (Supplementary Figure S7). Because of several missing atoms in the deoxyribose moieties of the terminal nucleotides, we analyzed the helical curvature of the internal 28-bp instead of the entire 34-bp in the complex of yeast topoisomerase II. Curvilinear fitting on this region gave rise to 140.3° (Supplementary Figure S7), comparable to its suggested degree of bending. We next analyzed the helical curvature of the 18-bp compartment enclosing the two scissile bonds, and we found a sequential increase in the curvature from the drug-bound (hTopIIβ-tc), the ‘resealed’ (sTopIV), to the gate-DNA in the non-covalent complex (yTopII-pcc; Figure 3). This curvature in the drug-unbound cleavage complex (hTopIIβ-cc; UD1) fluctuated during the simulation; however, we observed a population shift of the bending angle of the cleaved DNA toward that in the complex of the ‘closed’ DNA gate (Figure 3B). The invariant Ile172 of *E. coli* topoisomerase IV has been demonstrated to be necessary for DNA bending and cleavage (84). In our simulation, the equivalent Ile872 of human topoisomerase IIβ anchored in the base-pairing compartments of the +8/−4 and the +9/−5 nucleotides (animation S1), agreeing with the observations in crystallographic and biochemical studies.

### Coordinated inter-domain motion of topoisomerase II revealed the stratagem in conducting DNA cleavage and religation

The enzyme•DNA intermediate used in the current simulations carries two of the inter-subunit interfaces, the DNA gate and C gate. DNA gate in the cleavage core of the enzyme comprises the metal-binding TOPRIM domain and the nucleotide-binding WHD of each subunit. Wendorff *et al*. proposed a controlled association/dissociation mechanism between the DNA gate and the C gate through comparative structural analyses on the relative rotational status of the two WHDs (82). On the basis of this proposed demarcation, we examined the transition with respect to the configuration of the two WHDs.

#### Relative motion of winged-helix domains in topoisomerase II toward the complex with closed interface in the cleavage core

**Figure 4.**
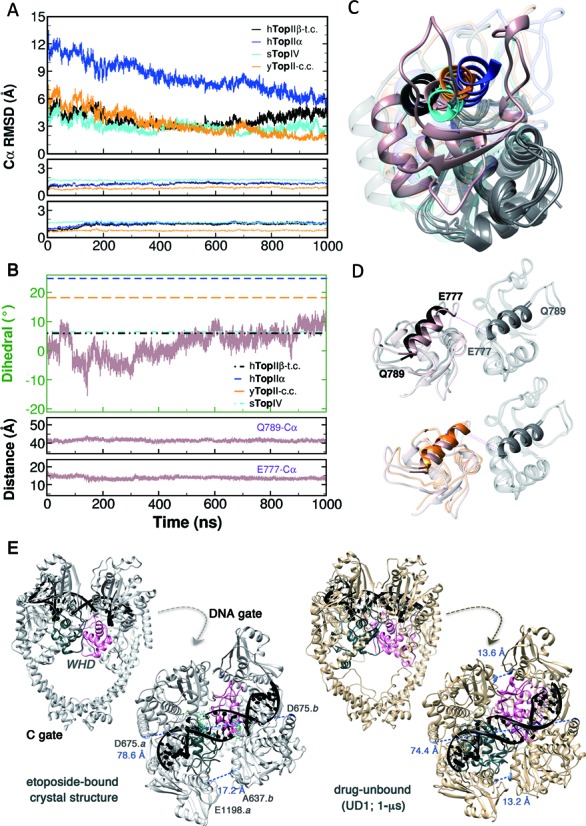
Inter-subunit movement of WHD in simulation UD1 toward the closed configuration of DNA gate. (**A**) RMSD between Cα atoms of WHD in the simulation with reference to the crystal of drug-bound complex (hTopIIβ-t.c.; 3QX3), the non-covalent complex of IIα isozyme (hTopIIα; 4FM9), topoisomerase IV with the ‘resealed’ DNA (sTopIV; 3KSB), and the cleavage complex of yeast enzyme (yTopII-c.c.; 3L4K). RMSD by pre-aligning WHD in the first protomer and comparing the dimeric arrangement of WHD *with respect to the counterpart* are plotted in the top panel; RMSD of aligning individual WHD in the same protomer are shown in the lower two panels. The moving averages of RMSD were calculated with a window size of 20 from 10^5^ snapshots. (**B**) transition of the dihedral (top) and inter-subunit distances (lower panels) between Cα atoms of Gln789 and Glu777 in the two protomers. The moving average of dihedral was calculated with a window size of 10 from 10^5^ snapshots. (**C** and **D**) Representative snapshot from the simulation (pink) revealing the relative movements of WHDs, with this domain in the first protomer (gray ribbon) pre-aligned to the reference crystal structures. The color scheme in A–D is consistent for the unaligned WHD. (**E**) comparison of the drug-bound crystal (hTopIIβ-t.c.) and the 1-μs snapshot revealed a ‘closing’ movement of the DNA gate. The distance between E1198.*b* and A637.*a* of the etoposide-bound complex is not applicable because of the missing Cα atom of A637.*a* in the crystal structure. Drug molecules are shown in light green.

To investigate predisposition of the enzyme in the drug-unbound complex (UD1) toward religation-compliant state, we selected crystal structures of the streptococcus topoisomerase IV with resealed DNA (sTopIV), the yeast topoisomerase II *cleavage* complex (yTopII-cc), and the pre-cleavage complex of human topoisomerase IIα (hTopIIα-pcc) as exemplars. Structural alignment of the drug-unbound IIβ complex to a single WHD of these structures stayed at the ground level, reflecting the well-preserved arrangement of this domain among eukaryotic and prokaryotic type II topoisomerases (Figure 4A, lower panels). RMSD values of inter-WHD alignment reveal transition of the WHD-WHD arrangement toward the exemplar structures (Figure 4A-top and C). The deviation from sTopIV soon reached a limitation of 3 Å, probably due to the higher sequence variety between human Top II and bacterial Top IV. Notably, the inter-WHD deviation from that adopted in the yeast cleavage complex diminished to the inherent difference of aligning a single domain. Inspired with the comparative analyses on rotational movements (82), we inspected the dihedral angle comprising Cα atoms of the Gln789–Glu777•Glu777–Gln789 residues and found temporal transition corresponding to the inter-subunit RMSD (Figure 4B and D). The simulation time required to observe reorienting of this dihedral is reminiscent of the time required for stabilizing the gate-DNA in *B-A-B* configuration (Figure 2), presumably indicating the concerted conformational transition in the enzyme and the substrate DNA.

In the current analyses, the hTopIIα pre-cleavage complex is the only exemplar with the DNA gate closed while the C gate open. It was proposed that in the configuration of DNA cleaving, the rotational status of WHDs would facilitate C gate closure, while resealing of the gate-DNA would render the WHD arrangement prompting C gate dissociation (82). On the other hand, the DNA gate was reported to undergo an opening of approximately 21 Å in Drosophila topoisomerase II with the substrate DNA of 28-bp, and the conversion rate constants for DNA gate opening and closing were reported to be 1–2 s^−1^ (20). In the current simulation, we observed a closing of the cleavage core by 4 Å (Figure 4E) and a torsional transition of the WHDs by more than 10° in the DNA gate (Figure 4B and D), which may not be sufficient to prompt apparent opening of the C gate. Although we did not observe C gate opening within the 1-μs simulation (Figure 4E), we observed a steady decrease in the deviation of the inter-subunit arrangement with respect to hTopIIα, the non-covalent complex with the open conformation of this C-terminal interface (Figure 4A-top and C). In addition, we also observed a consistent decrease in the Cα—Cα distance of the two intercalating Ile872 residues in the four simulations of the drug-unbound complex (Supplementary Figure S8, UD1–4), revealing an early sign in the closure of DNA gate. On the basis of our observation, the coordinated dynamics between DNA gate closure and C gate opening could be investigated in future simulations with longer time or enhanced sampling methods.

#### Restoration of the metal-binding motif for topoisomerase II-mediated DNA religation

**Figure 5.**
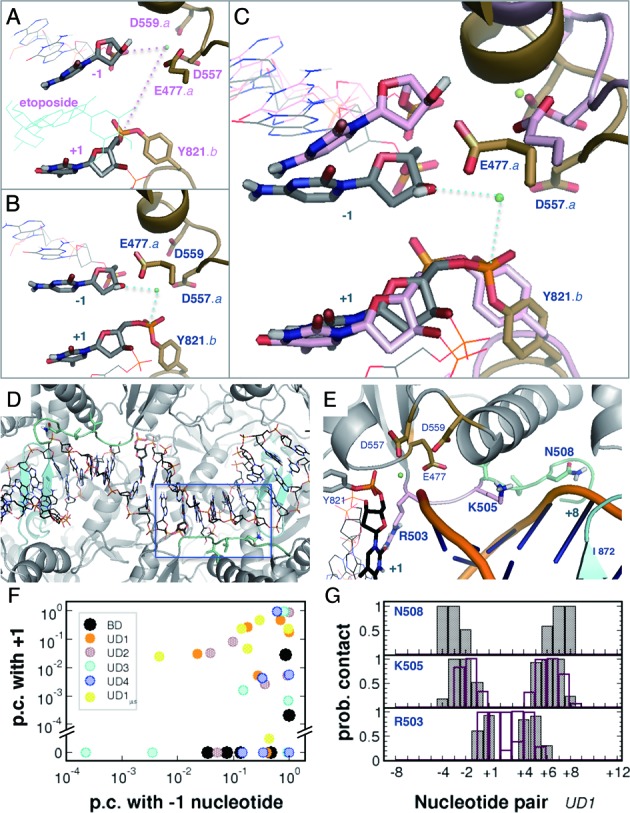
Interactions between topoisomerase II and the gate-DNA. Cleavage site and the Mg^2+^-binding motif in the 200th-ns snapshots from simulations BD (**A**) and UD1 (**B**), as designated in Table 1. The BD conformation is superimposed as pink drawing in (**C**), with the drug molecule hidden for clarity. (**D** and **E**) (flipped and enlarged), the conserved I506-N508 (green) in contact with backbone of the nucleotides flanking the base-pair intercalating I872 (cyan). (**F**) *In-trans* contact probabilities of Glu477, Asp557 and Asp559 with the −1 and the +1 nucleotides. The EDxD motifs of the two protomers in each simulation are displayed in the same color. Distribution of the 50–200 ns period of simulation UD1 (orange) is comparable to that of the 50–1000 ns trajectory (yellow; *UD1_μs_*). G, *in-cis* (filled) and *in-trans* (open bar) contact probabilities of Arg503, Lys505 and Asn508 with the nucleotide pairs in simulation UD1. Designation of the nucleotides is consistent with Figure 2.

The observed ‘recovering’ of the cleavage complex from the drug-entrapped conformation intrigued us to explore the interactions between the enzyme and the DNA. To this end, we conducted 200-ns molecular dynamics simulation of the drug-bound (BD) complex for comparison with the simulations of the drug-unbound (UD) complex, from which the initial 200-ns period was used (Table 1 and Supplementary Figure S9). We first mapped the probability of enzyme•DNA contacts with respect to the two polypeptide chains and the two oligonucleotide strands, giving rise to four pairing combinations as the 2-dimensional embedding of the contact configurations (Supplementary Figure S9, middle). The general patterns of direct contacts between topoisomerase II and the gate-DNA are comparable in the two systems. In the drug-unbound (UD) complex, however, we observed slightly increased contact probabilities between the metal-binding Glu477, Asp557 and Asp559 of one peptide chain and the cleavage site of its *complementary* oligonucleotide strand (Figure 5A–C, F, and Supplementary Table S3). Namely, as the cleaved-apart nucleotides with its bonded tyrosine in protomer *B* were approaching the sealing-compliant distance, the Mg^2+^-binding E•DxD motif of protomer *A* also approached the juxtaposition of this cleavage site, presumably to restore the active-site configuration in support of the flickering opportunities for the enzyme-mediated religation (Figure 5A–C and Supplementary Figure S3) (32,48). Such ‘cooperation *in trans*’, i.e. the synergy between the metal-binding acidic residues and the catalytic tyrosine of anther chain, in the cleavage of DNA has been demonstrated with the heterodimeric mutants of yeast topoisomerase II, in which one protomer contains an alanine-substituted mutation of the acidic residue at issue, while the other contains a tyrosine-to-phenylalanine mutation (85).

Another noteworthy feature resides in the contact configurations between DNA and the conserved Arg503-Asn508 (RGKILN) region. The polar side chain of Asn508 retained persistent contacts with the phosphodiester backbone of the nucleotides flanking the intercalating Ile872 (Figure 5E/G and Supplementary Figure S10). These nucleotides locate in the strand bonded with the catalytic tyrosine in the same protomer of the Asn508 in contact and the adjacent Ile872, rendering plausible cooperation *in cis*. In contrast, Lys505 and Arg503 appeared to swing around the base-pairing moieties enclosing the cleavage sites, and we observed diminished *in-trans* contact of Arg503 in the BD complex (Supplementary Figure S10). The R503E mutation has been found to reduce the DNA cleavage activity of topoisomerase IIβ (86), and the side chain rotamer of this residue have been demonstrated to adopt different conformations in response to the binding of different topoisomerase poisons (49,57). The diminished contact in the BD complex reiterates the involvement of Arg503 in the DNA cleavage of topoisomerase II. On the other hand, although mutation at the residue equivalent to Asn508 (N480A) was shown to inactivate the yeast topoisomerase II, the residue did not appear to assist the catalytic tyrosine in DNA cleavage and rejoining (85), leaving the functional role of this essential residue elusive in the past few decades. The invariant contacts between Asn508 and the intercalated nucleotides observed in the current study suggest the participation of this residue in supporting Ile872 to stabilize the DNA substrate for enzymatic operations.

#### ‘Accordion-playing’ of topoisomerase II: correlated enzyme•DNA motions accompanying nucleic acid transformation

**Figure 6.**
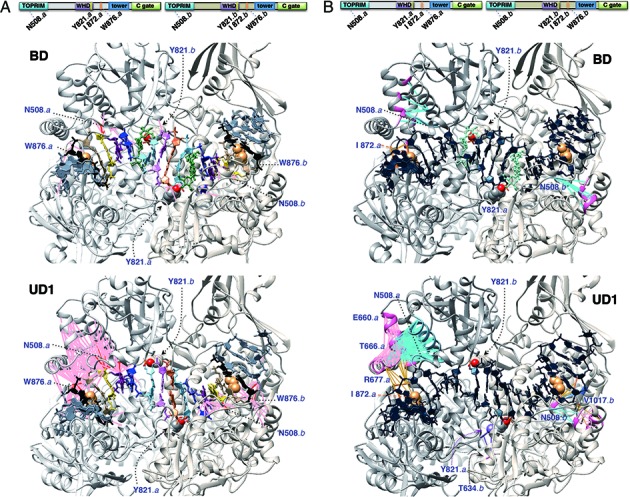
Correlated backbone motions in the topoisomerase II•DNA complexes. The catalytic Tyr821 and the DNA-intercalating Ile872 (orange) are shown in sphere representation. Drug molecules are shown in green. (**A**) Correlated motions between the enzyme and the gate-DNA. Pairwise correlations above the threshold of 0.8 and 0.7 are depicted as red and pink pseudo-bonds, respectively. The −5 to +9 nucleotide-pairs are colored in consistent scheme with Figure 2B. (**B**) Correlated inter-domain motions of topoisomerase II. Protein residues with nucleotide-correlated motions and concomitant correlations with different protein domains are depicted with the threshold of 0.7. Pairwise correlations between TOPRIM and the pink linker are colored in blue; correlations between the linker and the DNA-intercalating motif are in orange.

To further discriminate pivotal residues in the enzyme-conducted interface closing, we analyzed correlations between the backbone movements of the enzyme and gate-DNA, and we detected substantial correlated motions in the simulations of the drug-unbound (UD) complex (Supplementary Figure S9 and Table S4). As depicted in the molecular graphics with a threshold value of 0.7 (Figure 6A, UD1), pairwise correlated motions revealed a network covering TOPRIM and the following linker region, as well as the DNA-intercalating region (Ile872-Trp876), on one protomer of the enzyme with the −6/+10 to −2/+6 nucleotides accommodated in this compartment. In contrast, the counterpart protomer displayed merely countable correlated protein•DNA motions, reminiscent of the non-concerted fashion of topoisomerase II-mediated DNA religation (26). Interestingly, these correlated enzyme•DNA motions occurred on the nucleotides flanking one side of the middle segment that underwent *B*-to-*A* transition (−2 to +6), evocative of ‘squeezing’ the central bellows in playing an accordion. On the other hand, the backbone-interacting Asn508 displayed consistent *in-cis* correlated motions with the +7 nucleotide, in spite of the presence/absence of drug molecules (Figure 6A and Supplementary Table S4). This residue also retained correlated motions with the −3/+7 and −2/+6 nucleotide pairs in the succeeding 800-ns simulation of UD1 (not shown), concordant with the persisting fluctuations on these loci in ‘fine-tuning’ the cleavage-site enclosing segment to *A*-form (Figure 2B, *x*-disp).

#### Indispensable role of the linker preceding WHD in coordinating inter-domain motions of topoisomerase II and nucleic acid transformation

Apart from the conformation transitions of gate-DNA in the cleavage complex, substrate binding has also been observed to trigger inter-subunit rearrangements of the dimeric enzyme (22). We investigated pairwise correlations among the residues with apparent nucleotide-correlated motions and depicted those displaying concomitant correlations with residues in different domains (Figure 6B). Accordant with the distribution of correlated protein•DNA motions, we observed inter-wound correlations among individual motifs in the same enzyme protomer of the drug-unbound complex (UD1), connecting the movements of the conserved KILN^508^ and helix B'α4 (18) of TOPRIM, the linker between TOPRIM and WHD, and the DNA-intercalating motif. Although we also detected correlations between residues His775-Gly776 in WHD and the -1 nucleotide-pair, we did not observe concurrent correlations between these residues and the above-mentioned motifs; instead, the linker situated between TOPRIM-WHD displayed considerable correlations with helix B'α4 of TOPRIM, residues flanking the base-pair intercalating Ile872, and the Asn508 in contact with nucleic acid backbone. Notably, this linker region was disordered and could not be determined in the crystal of apo-form topoisomerase II, but it folded into a three-helix bundle in the crystal of DNA-bound enzyme, rendering extensive contacts with both DNA and the rest of the protein (22).

To verify the importance of the helix-bundle linker (residues 659–681) in coordinating the motion of the cleavage core, we determined the occurrence of such pairwise correlated motions with the threshold of 0.7 for the simulations UD1–4 and BD. The linker displayed more correlated inter-domain motions than the BD in three out of the four UD simulations (Supplementary Table S4), especially with the DNA-intercalating motif. Furthermore, all of the simulations revealed the lack of direct correlations between the intercalating motif with either helix B'α4 or the backbone-interacting KILN^508^ region, corroborating the liaison with these motifs by the linker. In a previous mutation-based screening for key residues of yeast topoisomerase II, Arg650 (equivalent to Arg677 in the linker of the IIβ) was demonstrated to be crucial to DNA cleavage/rejoining and was postulated to be present in the catalytic pocket, thereby interacting directly with the scissile phosphate (85). However, recent crystallographic structures of topoisomerase II could not support this hypothesis as this residue locates in the distant helix-bundle linker. The emergence of this linker region in our analyses not only echoes the dramatic conformational changes of topoisomerase II in response to substrate binding but also justifies the indispensable role of Arg677 in mediating the opening/closing of the DNA gate. We also noticed two additional residues with similar potentials in coordinating the movements of the metal-binding motif. Thr556 displayed consistent intensity of correlations with the adjacent Asp557 (0.91–0.96), the Asp559 (0.73–0.81) in close proximity, and the distant Glu477 (0.72–0.84) in one chain of the enzyme, regardless of the presence or absence of drugs. The other notable residue is the first one of helix B'α4, Ser563, which displayed considerable correlations with the three metal-binding residues (0.71–0.82) as well as the above-mentioned Thr556 (0.75–0.79) in three out of the four UD simulations but not the BD system. This serine residue demonstrated a concurrent correlation with the backbone-interacting Asn508 at an intensity of 0.65–0.79, suggesting a conceivable role in coordinating the motions of the metal-binding and the DNA backbone-binding motifs. The plausible functions of Thr556 and Ser563 await further investigations in the future.

In contrast to the TOPRIM and the operative linker, the backbone motions of WHDs displayed generally lower correlations, either with other motifs or in itself (Supplementary Figure S11). Such low correlations with respect to WHDs were also observed in analyses using the remainder of the microsecond simulation (not shown). An intuitive interpretation could follow the nature of rigid-body movements of a single WHD, as demonstrated in the ground-level intra-domainial RMSD (Figure 4A, lower panels) and the relatively low self-correlation. The *en bloc* movements between WHDs and TOPRIM domains have been observed in a comparison study using the crystal structures of the etoposide-bound IIβ isozyme and the drug-free IIα isozyme, and it was proposed that drug binding occurs during the transient opening, or ‘breathing’, of the DNA gate (82). Instead of the WHD, the helix-bundle linker between it and the preceding TOPRIM appeared to organize the collective motions of the metal-binding motif, the two distinct nucleotide-binding motifs, and the bound DNA substrate during the closure of DNA gate.

## CONCLUSION

Type II topoisomerase are essential enzymes in biological systems and prominent therapeutic targets. A wealth of structural and biochemical information have depicted the framework of the enzyme-conducted disentanglement of DNA double helices. However, the transitory nature of DNA cleavage and religation hindered further understanding of the dynamic architecture underlying these enzymatic operations. We exploited the crystal structures of the cleavage complex with religatable DNA substrate and conducted the microsecond atomistic molecular dynamics simulation to investigate the enzyme-mediated religation, with our newly derived charges for the 5′-phosphotyrosyl linkage. The drug-unbound intermediate displayed transitions toward the resealing-compliant configuration: closing distance between the cleaved DNA termini, *B*-to-*A* transformation of the double helix, and restoration of the metal-binding motif in the de-poisoned enzyme. The occurrence of sealing-compliant distance on one strand was not concerted with that on the complementary strand. In addition, the helical axis of the cleaved DNA appeared to bend to the configuration of resealed DNA in the complex. We also observed relative movements of WHDs toward the closed state of the DNA gate. It should be noticed that, however, in the drug-bound crystal structure used in the current work, there is only *one* metal ion at each cleavage site. The need for two metal ions in the topoisomerase-mediated cleaving/sealing reaction has been proposed from crystallographic and biochemical studies, and the binding of topoisomerase poisons was presumed to alter the disposition of metal ions and perturb the cleavage/religation equilibrium (32,82,87). Although the configuration in the current work is different with regard to the number of metal ions *in situ*, the coordination of the retained crystallographic Mg^2+^ could be preserved via water molecules surrounding the cleavage site. Furthermore, the increased probability of contacts between the Mg^2+^-binding E•DxD motif and the cleavage site suggested the inclination of topoisomerase II to restore the active-site configuration for DNA religation. Instead of stressing an ‘exact’ reactant state for the chemical reaction achieved with the use of molecular mechanics force field, we demonstrated the *slow* conformational transitions toward the closed-interface configuration that can hardly be observed in picoseconds of QM/MM simulations. Our microsecond simulation rendered a conceivable picture of the reversible, double-strand cleaving/resealing equilibrium conducted by topoisomerase II.

While correlated motions between protein and nucleic acids have been reported in *trp*-repressor (88), Ets-1 transcription factor (89,90), and the monomeric topoisomerase IB (34), our work is the first to reveal correlated protein•DNA motions from hundreds-of-nanosecond simulations of such a large system. We demonstrated the participation of the helix-bundle linker preceding WHD in coordinating the movements of the metal-binding TOPRIM, the nucleotide backbone and base-pair binding motifs, and the bound nucleic acid substrate during the opening/closing of the DNA gate. We also enlightened the indispensible function of Arg677 in the rejoining of DNA, which could not be supported by crystallographic information. In addition to the correlation analyses, we determined the probabilities of protein-DNA contacts that do not entail structural alignment for the ensemble of conformations. With the use of this distance-based approach in mapping the contact configurations between the enzyme and the DNA, we identified the essential role of the backbone-binding Asn508, which also displayed consistent correlated motions with the interacting nucleotides.

The current simulations of the drug-bound and the drug-unbound enzyme•DNA intermediates revealed the coordinated conformational transitions of the enzyme and the DNA. The operative linker between TOPRIM and WHD organized the motions of the cleavage core and the double-stranded DNA during the closing of cleavage sites and the *B*-to-*A* transitions. As the bound compartment of DNA was settled, presumably in favour of the rejoining operation, subsequent rearrangements in the torsional state of WHDs drived the DNA gate to a closed configuration that could facilitate the opening of the C gate for strand passage. We observed a nearly vectorial transition in the restoration of the enzyme from the ‘poisoned’ state to the configuration compliant for DNA religation and identified the previously uncharacterized roles of Asn508 and Arg677 critical in the process. Our findings delineate the dynamic mechanism of the DNA religation conducted by type II topoisomerases.

## Supplementary Material

SUPPLEMENTARY DATA
