# Supplementary material for: Recovery of the poisoned topoisomerase II for DNA religation: coordinated motion of the cleavage core revealed with the microsecond atomistic simulation
Source: Nucleic Acids Res. 2015 Jul 6;43(14):6772–86. doi: 10.1093/nar/gkv672 (PMC4538842; doi:10.1093/nar/gkv672)
Supplement: SUPPLEMENTARY DATA [file supp_43_14_6772__index.html]

Recovery of the poisoned topoisomerase II for DNA religation: coordinated motion of the cleavage core revealed with the microsecond atomistic simulation — Recovery of the poisoned topoisomerase II for DNA religation: coordinated motion of the cleavage core revealed with the microsecond atomistic simulation — SUPPLEMENTARY DATA 

# Recovery of the poisoned topoisomerase II for DNA religation: coordinated motion of the cleavage core revealed with the microsecond atomistic simulation

## SUPPLEMENTARY DATA

- SUPPLEMENTARY DATA
- SUPPLEMENTARY DATA
