# Supplementary material for: Recovery of the poisoned topoisomerase II for DNA religation: coordinated motion of the cleavage core revealed with the microsecond atomistic simulation
Source: Nucleic Acids Res. 2015 Jul 6;43(14):6772–86. doi: 10.1093/nar/gkv672 (PMC4538842; doi:10.1093/nar/gkv672)
Supplement: SUPPLEMENTARY DATA [file supp_gkv672_nar-01057-f-2015-File009.pdf]

Recovery of the poisoned topoisomerase II for DNA religation: coordinated motion of the cleavage core revealed with the microsecond atomistic simulation

Nan-Lan Huang[1], Jung-Hsin Lin[1,2,3,*]

[1] Research Center for Applied Sciences, Academia Sinica, Nangang, Taipei, 11529, Taiwan
[2] Institute of Biomedical Sciences, Academia Sinica, Nangang, Taipei, 11529, Taiwan
[3] School of Pharmacy, National Taiwan University, Taipei, 10050, Taiwan

* To whom correspondence should be addressed. Tel: 886-2-27873143; Fax: 886-2-27873211; Email: jlin@ntu.edu.tw

SUPPLEMENTARY INFORMATION

METHODS

Molecular models
Molecular dynamics simulations
Modified force field parameters for the 5'-phosphotyrosyl bond of topoisomerase II cleavage complex
Molecular dynamics simulations on the unbound DNA double helix with the 5'-phosphotyrosyl bonds
Correlation analyses of the drug-bound and the unbound complexes

RESULTS

Test simulation on the unbound DNA
Simulation of the cleavage complex using the drug-free crystal structure

DISCUSSION

Coordination of metal ions and the cleavage-site configuration in topoisomerase II•DNA intermediate

TABLES and FIGURES

Table S1.
Table S2.
Table S3.
Table S4.

Figure S1.
Figure S2.
Figure S3.
Figure S4.
Scheme S1.
Figure S5.
Figure S6.
Figure S7.
Figure S8.
Figure S9.
Figure S10.
Figure S11.

Legend of Animation S1.

METHODS

Molecular models. The crystal coordinates of the etoposide-stabilized (PDB code: 3QX3) and the eluted drug-free (PDB code: 4J3N) cleavage complex of the human topoisomerase IIβ were used. Atomic coordinates of the missing amino acid residues were assigned by aligning the template structure generated with the threading-based ITASSER program (1-3). Notably, the program constructed Val594-Lys630 to be a “Greek Key”-like motif, probably based on the sequence homology with the corresponding region of the IIα isozyme. This TOPRIM-associated Greek-key motif was observed in the crystal structure of topoisomerase IIα (PDB code: 4FM9), but the building residues were missing from the crystal structure of the drug-bound complex of topoisomerase IIβ. In the paper of the former structure, the authors assumed the absence of this motif in the latter structure to result either from the drug-induced conformational changes or from the lack of interaction with the subtype-specific residues following helix A’α11 of the IIβ isozyme that were also missing from the structure (4). We found the “Greek Key”-like motifs modeled in the drug-unbound (*UD*) complex of topoisomerase IIβ turned into poorly folded loops during the simulation, disagreeing with the first assumption and providing a provisional support of the second assumption. Atomic coordinates of the missing nucleotide in the drug-free crystal structure were assigned by alignment with the phosphodiester backbone of its complementary strand. The amino acid residues were protonated with the use of PDB2PQR (5,6), and the nucleotides were protonated using the LEaP program of the AMBER package (7). Each of the two cleavage complex systems comprises 24,186 atoms of the homodimeric topoisomerase cleavage core and C gate (Lys452-Asp1201), 1266 atoms of the 20-bp gate-DNA, the crystal Mg^{2+} and the crystal water molecules.

Molecular dynamics simulations. The topology files and parameter files were constructed, with the ff99SB force field for protein (8,9) and parmbsc0 modifications for nucleic acid (10), using the LEaP module of AMBER 14. For the phosphotyrosyl bond on the 5’-terminus in the topoisomerase II cleavage complex, we employed two-stage RESP fitting to obtain atomic charges, based on the crystal coordinates of the covalently linked Tyr821 and the +1 nucleotide. Each system was solvated with explicit water molecules in a orthogonal box, with the buffer distance of 12.0 Å to the box boundary and the closeness parameter of 0.75 Å, using the TIP3P solvent model (11). Ions were supplemented using the addIons module to neutralize the system and to simulate an environment of 150 mM sodium chloride aqueous solution. Each system contains more than 171,101 atoms in sum. Molecular dynamics simulations were carried out using the PMEMD module, with the use of particle-mesh Ewald (PME) method for calculating full electrostatic interactions of a periodic box in the macroscopic lattice of repeating images. Energy minimizations were carried out with restraints on atoms of the amino acids, the nucleotides, the crystallographic Mg^{2+} and water molecules. The systems were heated to 310 K and regulated using the weak-coupling algorithm with the coupling time constant of 0.2 ps. System pressures were regulated toward 1 bar using isotropic position scaling, and the Berendsen barostat was used with the relaxation time of 2 ps. Isothermal-isobaric simulations were conducted to equilibrate the system densities for 1 ns with the time step of 1 fs. Production simulations were carried out with the time step of 2 fs and with SHAKE constraints (12) on the bonds not involving hydrogen. Simulation of each system using a graphical processing unit gave

rise to an efficiency of about 9.61 ns/day. The main simulation was carried out for 1000 ns. Sampling of individual snapshots of the production simulations were carried out using the cpptraj module.

Modified force field parameters for the 5'-phosphotyrosyl bond of topoisomerase II cleavage complex. The crystal coordinates of the covalently linked Tyr821-dT(+1) in the etoposide-stabilized complex (PDB code: 3QX3) were used to derive atomic charges of these residues. Tyr821 was capped with ACE/NME and the O3' of dT(+1) was capped with a methylphosphate group using the LEaP program of AMBER package. Charge derivation was conducted using Gaussian 03 (13). Geometry optimization of the entire molecule was carried out at B3LYP/6-31G* level, and the electrostatic potential was calculated at the HF/6-31G* level. Subsequently, two-stage RESP (14) fitting was carried out, with the equivalent hydrogen atoms of each group constrained to be identical and the capping groups constrained to previously determined values. The newly derived charges were demonstrated in Fig S1. To generate modified force field parameters for simulations of the systems including this non-standard residue, we retrieved compatible parameters from ff10 (15), phosaa10 (16), and parmbsc0 (10). The tyrosyl moiety and the thymidinyl moiety were defined base on the corresponding standard residues, yet with the O…P bonding atoms linkable upon generating topology files using the LEaP module.

Molecular dynamics simulations on the unbound DNA double helix with the 5'-phosphotyrosyl bonds. To test the validity of the modified force field parameters for the phosphotyrosyl bond on the 5'-end of the dT(+1) residue, we set the gate-DNA apart by removing all the other enzyme residues except the two bonded Tyr821. We then capped the retained Tyr821 residues with ACE/NME and generated the topology and parameter files on the entire molecule. Molecular dynamics simulations were carried out with the *parmbsc0* and the newly generated modified parameters for 200 ns.

Correlation analyses of the drug-bound and the unbound complexes. The 50-200 ns periods of the simulations on the drug-unbound complexes (UD1-UD4) were selected to compare with that of the drug-bound complex (BD; Table 1). Each trajectory was sampled at an interval of 100 ps for the following analyses. The fitting of an ensemble of conformations from each trajectory was carried out by structural alignment with respect to the Cα atoms of the regions with well-folded secondary structures in its reference structure. Correlation analyses were carried out according to Lange and Grubmüller (17) using the g_correlation module of gromacs-3.3.3 with the *nofit* and the *linear* option, since this module is not implemented in more recent versions of gromacs. The method is based on mutual information and gives rise to correlation coefficients in the range of zero and unity. The correlation coefficient assumes the value of 1 for perfectly correlated motions and vanishes for completely uncorrelated motions. The correlation analyses were conducted on the 1,500 Cα atoms of the protein and the forty C5' atoms of the DNA. We have tested the influence of reference structure on the correlation analyses by using the initial conformation, the last conformation, and the average structure of each trajectory as the reference for the fitting ahead of the analyses. The use of different reference structures in the preceding fitting did not result in a discrepancy in the functional interpretation of the correlation data. The use of the regions with well-folded secondary structures in

the preceding fitting may account for the consistent results of correlation analyses, since the structural alignment is insensitive to the interfering effects by insignificant fluctuations of the poorly folded regions. We demonstrated the results with the use of the average structure herein. The average structure of each trajectory was selected by a hierarchical agglomerative approach of clustering using the cpptraj module of AMBER 14. An initial clustering with an adequate epsilon value gave rise to several cluster centroids from the ensemble of conformations. These conformations were subjected to the second stage of clustering, and the ultimate centroid conformation served as the reference for the structural fitting in advance of the correlation analyses of the trajectory.

RESULTS

Test simulation on the unbound DNA

The double helix rapidly "relaxed" from the curved, crystal conformation into a linear *B*-DNA, with the helical bending of 5.5 º and the average Twist of 35.2 º (Fig S2). The tendency of solvated DNA molecules toward *B*-form is concordant with previous observations from simulations using AMBER force fields (18-21). The distances between the O3'(-1) and the P(+1) atoms of the cleaved-apart nucleotide steps fluctuated drastically and frequently approached a lower limit of 3 Å (Fig S1).

Simulation of the cleavage complex using the drug-free crystal structure

O3'(-1)—P(+1) distances of the gate-DNA.

In addition to the simulations starting from the crystal conformation of the drug-stabilized complex, we conducted another distinct simulation using the crystal of drug-free topoisomerase IIβ•DNA intermediate which was prepared by eluting the drug molecules from an aliquot of the ternary complex (22) (Scheme S1). In contrast to the slow DNA resealing observed in the drug-free bacterial topoisomerase IV (23), the cleaved-apart ends in the topoisomerase IIβ intermediate did not appear to move closer; moreover, one of the +4 nucleotides was missing from the determined structure, likely due to the pliability after drug elution. We reconstructed this missing nucleotide and carried out simulation of this drug-free intermediate. Even though it took longer simulation time (about 40 ns), we could still observe the O3'(-1)—P(+1) distance on one strand came toward the resealing-compliant value for several times (Fig S5), in agreement with the simulations conducted by manually removing drug molecules from the drug-stabilized crystal complex.

Nucleic acid conformations.

We noticed that 200-ns of the main simulation was not sufficient to settle the gate-DNA in the *B-A-B* configuration (Fig 2); instead, we compare the temporal transition of Slide and *x*-displacement (*x*-disp) of individual base-pair/steps to the initial 200-ns of the simulation on cleavage complex *by excluding drug molecules from the drug-bound crystal* (Scheme S1). The trends in Slide and *x*-disp are comparable along the simulations of the drug-free and the drug-unbound complexes, except for the +1/+2 and the +4/+5 steps (Fig S6). Similar to the need for longer simulation time to approach the

religation-compliant O3'(-1)—P(+1) distance using the drug-free crystal structure, the discrepancy in these two base-pair steps may reflect the susceptibility of molecular dynamics simulations to the selection of starting conformation.

DISCUSSION

Coordination of metal ions and the cleavage-site configuration in topoisomerase II•DNA intermediate

With the use of molecular mechanics force fields, we obtained the conformation with the O3'(-1)—P(+1) distance of 3.18 Å, by using the sampling frequency of 1 ps^{-1} during the microsecond simulation. Recently, two distinct computational studies were carried out, based on the crystal structure of yeast topoisomerase II cleavage complex which was determined with the use of phosphorotholate DNA substrate and Zn^{2+}, to investigate mechanisms of the cleavage and the religation, respectively (24,25). The distance between the tyrosine-linked phosphorus and the O3' atom on its symmetric neighbour unit is 3.99 Å in the reference crystal structure. The sulphur atom was displaced by oxygen, and the two zinc ions at the catalytic site were displaced by Mg^{2+} in their constructed models. QM/MM geometry optimization of one such model reached the "reactant" state for the *sealing reaction*, with the O3'(-1)—P(+1) distance of 2.9 Å, and subsequent search for reaction pathway achieved the "product" state with this forming bond length of 1.8 Å. In the other work, QM/MM optimization also brought the equivalent model to the non-covalent complex for investigating the *cleavage reaction*, with the bonded O3'(-1) situated around 1.6 Å away from P(+1), and with the tyrosine detached from the +1 nucleotide. It should be noticed that, however, in the crystal structure used in these studies, the "catalytic region" possessed *two* metal ions, while in the drug-bound crystal structure used in the current work, there is only *one* metal ion at each cleavage site (Fig S3). The need for two metal ions in the topoisomerase-mediated cleaving/sealing reaction has been proposed from crystallographic and biochemical studies, and the binding of topoisomerase poisons was presumed to alter the disposition of metal ions and perturb the cleavage/religation equilibrium (4,26,27). Although the configuration in the current work is different with respect to the number of metal ions *in situ*, the octahedral coordination of the retained crystallographic Mg2+ could be preserved via water molecules surrounding the cleavage site (Fig S3). The configuration achieved in the current work could serve as the commencing state for QM/MM study to address the specific issue on the mechanism of religation and subsequent events in the catalytic cycle after drug releasing, and the system should be supplied with magnesium ions at physiological level to simulate the environment where the entry of additional metal ion into the catalytic region is plausible (24).

REFERENCES

1. Zhang, Y. (2008) I-TASSER server for protein 3D structure prediction. *BMC Bioinformatics*, **9**, 40.
2. Yang, J., Yan, R., Roy, A., Xu, D., Poisson, J. and Zhang, Y. (2014) The I-TASSER Suite: protein structure and function prediction. *Nat Methods*, **12**, 7-8.
3. Roy, A., Kucukural, A. and Zhang, Y. (2010) I-TASSER: a unified platform for automated protein structure and function prediction. *Nat Protoc*, **5**, 725-738.

4. Wendorff, T.J., Schmidt, B.H., Heslop, P., Austin, C.A. and Berger, J.M. (2012) The Structure of DNA-Bound Human Topoisomerase II Alpha: Conformational Mechanisms for Coordinating Inter-Subunit Interactions with DNA Cleavage. *J Mol Biol*, **424**, 109-124.
5. Dolinsky, T.J., Czodrowski, P., Li, H., Nielsen, J.E., Jensen, J.H., Klebe, G. and Baker, N.A. (2007) PDB2PQR: expanding and upgrading automated preparation of biomolecular structures for molecular simulations. *Nucleic Acids Res*, **35**, W522-525.
6. Dolinsky, T.J., Nielsen, J.E., McCammon, J.A. and Baker, N.A. (2004) PDB2PQR: an automated pipeline for the setup of Poisson-Boltzmann electrostatics calculations. *Nucleic Acids Res*, **32**, W665-667.
7. Salomon-Ferrer, R., Case, D.A. and Walker, R.C. (2013) An overview of the Amber biomolecular simulation package. *Wiley Interdisciplinary Reviews-Computational Molecular Science*, **3**, 198-210.
8. Duan, Y., Wu, C., Chowdhury, S., Lee, M.C., Xiong, G., Zhang, W., Yang, R., Cieplak, P., Luo, R., Lee, T. *et al.* (2003) A point-charge force field for molecular mechanics simulations of proteins based on condensed-phase quantum mechanical calculations. *J Comput Chem*, **24**, 1999-2012.
9. Warshel, A., Papazyan, A. and Kollman, P.A. (1995) On low-barrier hydrogen bonds and enzyme catalysis. *Science*, **269**, 102-106.
10. Perez, A., Marchan, I., Svozil, D., Sponer, J., Cheatham, T.E., Laughton, C.A. and Orozco, M. (2007) Refinenement of the AMBER force field for nucleic acids: Improving the description of alpha/gamma conformers. *Biophysical Journal*, **92**, 3817-3829.
11. Jorgensen, W.L., Chandrasekhar, J., Madura, J.D., Impey, R.W. and Klein, M.L. (1983) Comparison of simple potential functions for simulating liquid water. *J Chem Phys*, **79**, 926-935.
12. Ryckaert, J.-P.C., G.; Berendsen, H.J.C. . (1977) Numerical integration of the cartesian equations of motion of a system with constraints: Molecular dynamics of n-alkanes. . *J Comput Phys*, **23**, 327–341.
13. Frisch, M.J. (2006) Optimizing large molecules with Gaussian 03. *Chemicke Listy*, **100**, A9-A9.
14. Wang, J.M., Cieplak, P. and Kollman, P.A. (2000) How well does a restrained electrostatic potential (RESP) model perform in calculating conformational energies of organic and biological molecules? *Journal of Computational Chemistry*, **21**, 1049-1074.
15. Homeyer, N., Horn, A.H., Lanig, H. and Sticht, H. (2006) AMBER force-field parameters for phosphorylated amino acids in different protonation states: phosphoserine, phosphothreonine, phosphotyrosine, and phosphohistidine. *J Mol Model*, **12**, 281-289.
16. Steinbrecher, T., Latzer, J. and Case, D.A. (2012) Revised AMBER parameters for bioorganic phosphates. *J Chem Theory Comput*, **8**, 4405-4412.
17. Lange, O.F. and Grubmuller, H. (2006) Generalized correlation for biomolecular dynamics. *Proteins*, **62**, 1053-1061.
18. Reddy, S.Y., Leclerc, F. and Karplus, M. (2003) DNA Polymorphism: A Comparison of Force Fields for Nucleic Acids. *Biophysical Journal*, **84**, 1421-1449.
19. Ricci, C.G., de Andrade, A.S.C., Mottin, M. and Netz, P.A. (2010) Molecular Dynamics of DNA: Comparison of Force Fields and Terminal Nucleotide Definitions. *The Journal of Physical Chemistry B*, **114**, 9882-9893.
20. Pérez, A., Lankas, F., Luque, F.J. and Orozco, M. (2008) Towards a molecular dynamics consensus view of B-DNA flexibility. *Nucleic Acids Research*, **36**, 2379-2394.
21. Beveridge, D.L., Cheatham, T.E. and Mezei, M. (2012) The ABCs of molecular dynamics simulations on B-DNA, circa 2012. *Journal of biosciences*, **37**, 379-397.
22. Wu, C.C., Li, Y.C., Wang, Y.R., Li, T.K. and Chan, N.L. (2013) On the structural basis and design guidelines for type II topoisomerase-targeting anticancer drugs. *Nucleic Acids Res*, **41**, 10630-10640.

23. Laponogov, I., Pan, X.S., Veselkov, D.A., McAuley, K.E., Fisher, L.M. and Sanderson, M.R. (2010) Structural basis of gate-DNA breakage and resealing by type II topoisomerases. *PLoS One*, **5**, e11338.
24. Palermo, G., Stenta, M., Cavalli, A., Dal Peraro, M. and De Vivo, M. (2013) Molecular Simulations Highlight the Role of Metals in Catalysis and Inhibition of Type II Topoisomerase. *J Chem Theory Comput*, **9**, 857-862.
25. Hanaoka, K., Shoji, M., Kondo, D., Sato, A., Yang, M.Y., Kamiya, K. and Shiraishi, K. (2014) Substrate-mediated proton relay mechanism for the religation reaction in topoisomerase II. *Journal of Biomolecular Structure & Dynamics*, **32**, 1759-1765.
26. Schmidt, B.H., Burgin, A.B., Deweese, J.E., Osheroff, N. and Berger, J.M. (2010) A novel and unified two-metal mechanism for DNA cleavage by type II and IA topoisomerases. *Nature*, **465**, 641-644.
27. Deweese, J.E., Burgin, A.B. and Osheroff, N. (2008) Human topoisomerase IIalpha uses a two-metal-ion mechanism for DNA cleavage. *Nucleic Acids Res*, **36**, 4883-4893.

SUPPLEMENTARY FIGURES

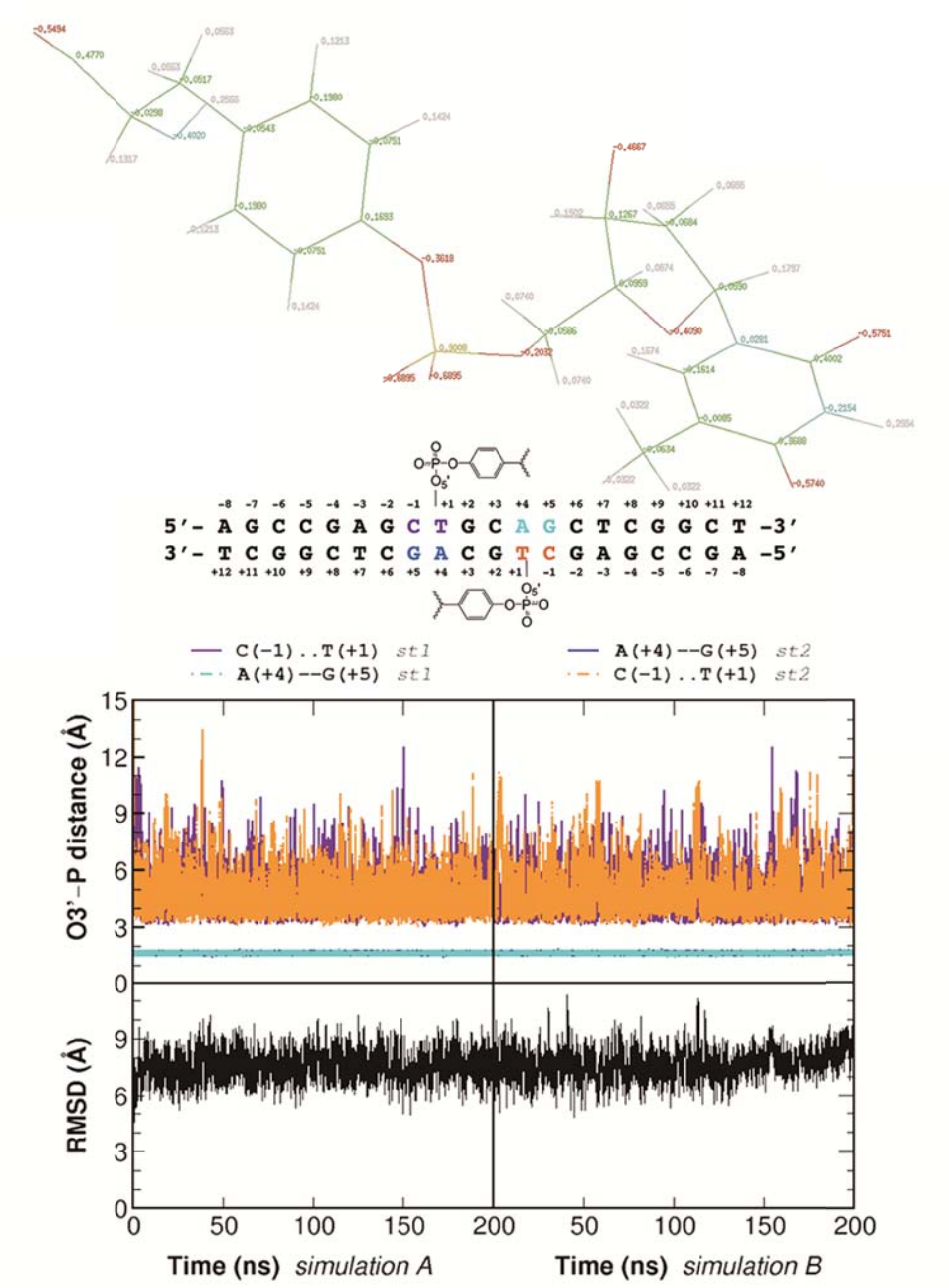

Figure S1. Atomic partial charges of the covalently bonded tyrosine and thymidine (upper), O3'-P distances and backbone RMSD in the test simulations of the *unbound* DNA (lower panel). As the control, the un-cleaved steps complementary to the cleaved ones remain stable throughout the 200-ns simulations (cyan and blue lines). The image of atomic charges was generated using xLEaP.

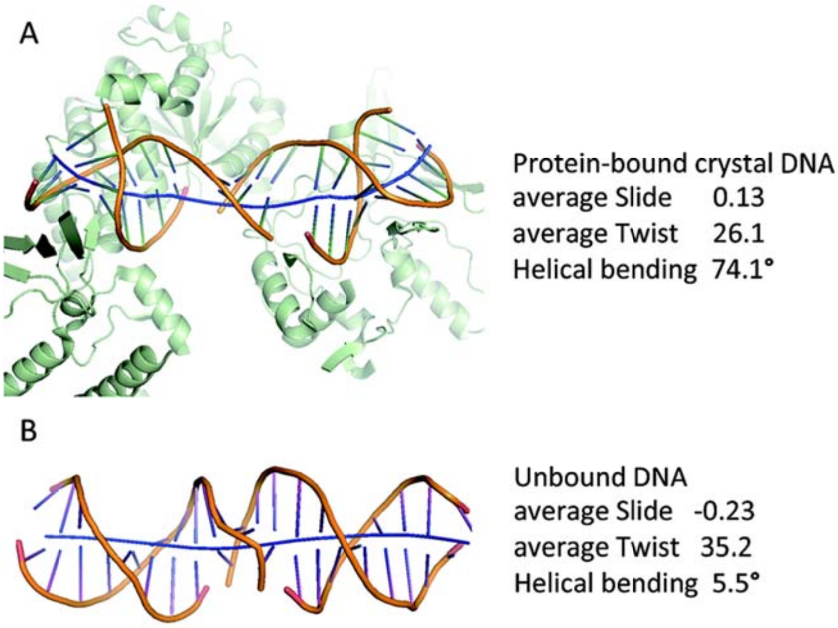

Figure S2. Nucleic acid conformations of the protein-bound DNA in the crystal (A; PDB code 3QX3) and the representative snapshot from simulations of the *unbound* DNA (B). The covalently bonded tyrosyl residues were hidden for better clarity on the cleaved nucleotide steps. Helical axes were generated using Curves+.

Table S1. Hess's cosine contents of the first three principal components in the simulations.

**Cosine contents**	**UD1**	**UD2**	**UD3**	**UD4**	**BD**
1st principal component	0.320	0.139	0.232	0.156	0.253
2nd principal component	0.106	0.291	0.418	0.517	0.057
3rd principal component	0.000	0.016	0.027	0.088	0.029

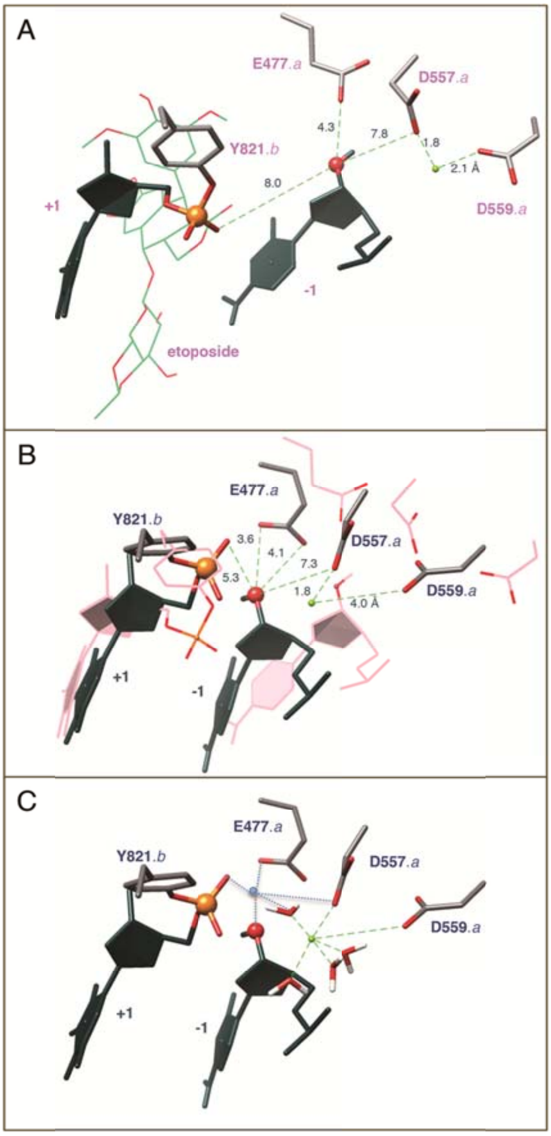

Figure S3. Cleavage site and the Mg^{2+}-binding motif in the 200th-ns snapshots from simulations of drug-bound (A) and drug-unbound (B) topoisomerase II complex, as indicated in Scheme 1 of the main context. The drug-*bound* conformation is superimposed as pink line-drawing in panel B, with the drug molecule hidden for clarity. C, octahedral coordination of the retained Mg^{2+} (green) with the metal-binding residues and surrounding water molecules. The conceivable location of the Mg^{2+} missing in drug-bound crystal was depicted in blue. Molecular graphics were generated based on the orientation of the “reactant” state proposed by Hanaoka *et al*. (25)

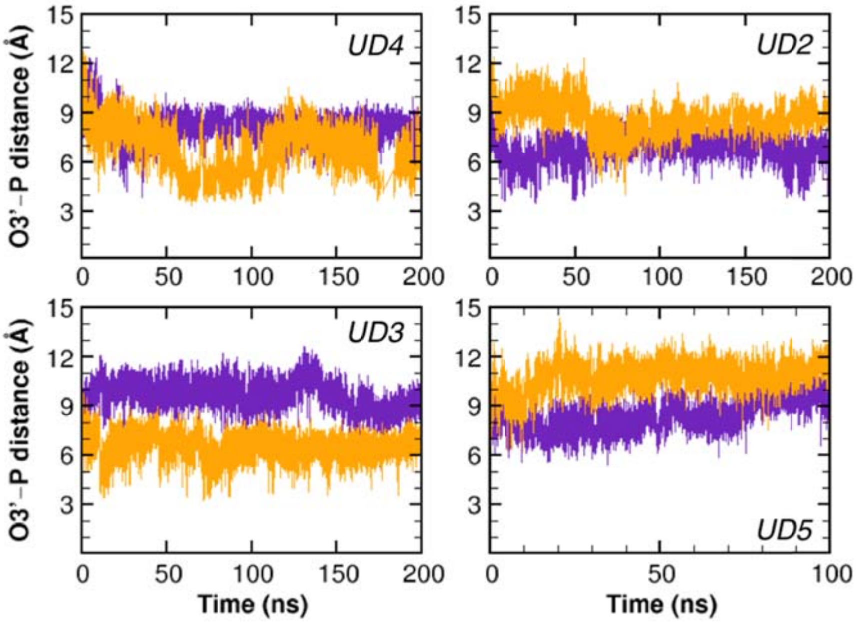

Figure S4. O3'-P distances of the cleaved-apart nucleotides on the two strands in the repeating simulations of drug-unbound topoisomerase II cleavage complex (UD2-5).

Table S2. O3'-P distances of the cleaved-apart nucleotides on the two strands in the simulations of drug-unbound topoisomerase II cleavage complex (UD1-5).

Systems	UD1	UD2	UD3	UD4	UD5 [b]
Complex type	Drug-unbound	Drug-unbound	Drug-unbound	Drug-unbound	Drug-unbound
Simulation length	1000 ns	200 ns	200 ns	200 ns	100 ns
**DNA O3'(-1) : P(+1) distance (Å)**					
Strand 1 (avg)	**5.88** ± 1.16	**6.86** ± 0.73	9.74 ± 0.81	8.21 ± 0.76	**8.47** ± 0.92
(min)	3.18	3.41	5.30	3.51	5.35
Strand 2 (avg)	7.36 ± 0.75	8.72 ± 0.88	**6.60** ± 0.70	**6.69** ± 1.54	10.82 ± 0.83
(min)	3.22	4.02	3.19	3.32	6.09
$p < 0.0001$ for the strand with the smaller average being indistinguishable from the complementary strand in the same complex [a]					

[a] The null hypothesis between the strand with the shorter and the longer average target distance in the same complex (simulations UD1-UD5) were tested using two-way analysis of variance (ANOVA).
[b] The UD5 simulation did not reveal apparent shortening of the distance and therefore was not included in the subsequent analyses.

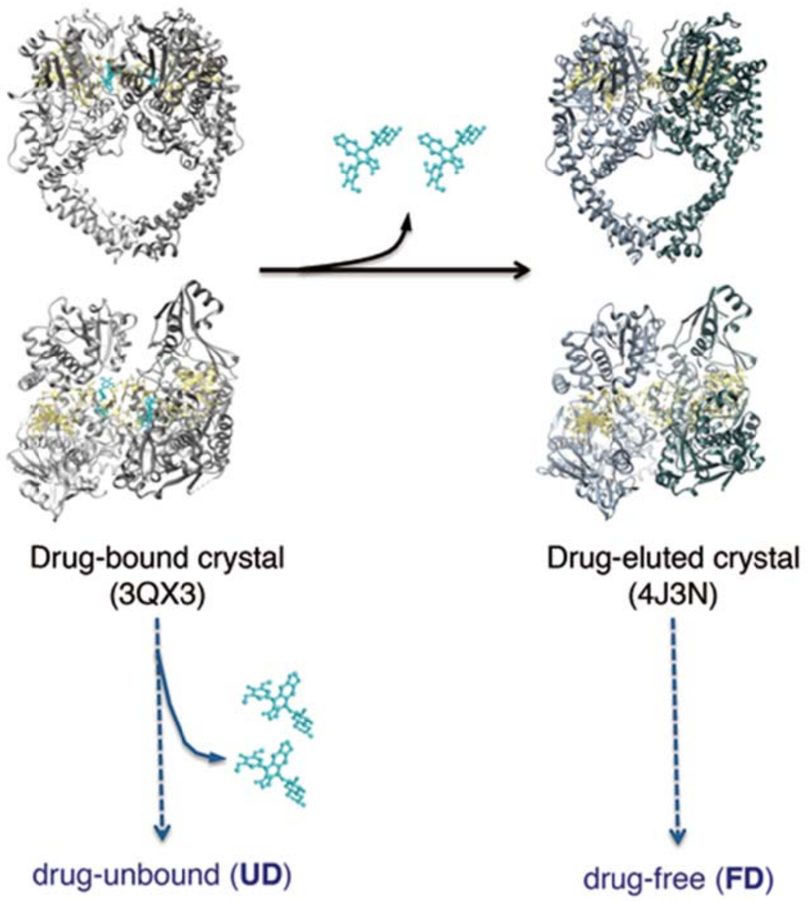

Scheme S1. Distinct simulations of the topoisomerase IIβ•DNA intermediates using different crystal structures as starting conformations.

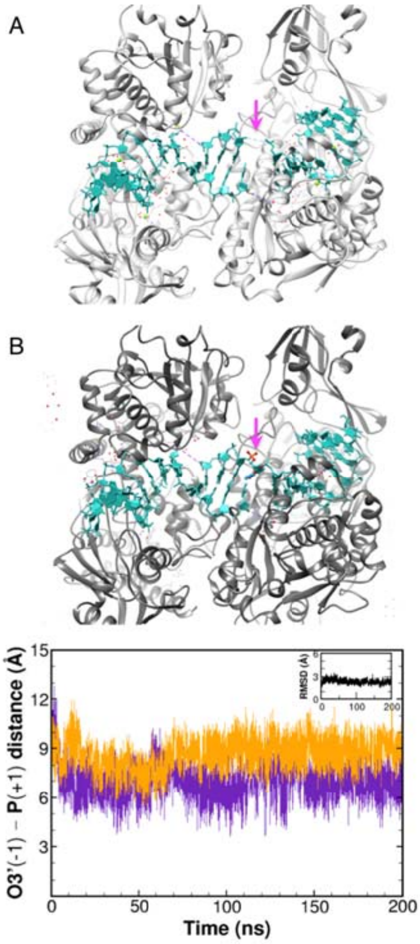

Figure S5. Crystal conformation of the *eluted drug-free* (FD) complex (A; PDB code: 4J3N) and molecular model with the missing +4 nucleotide (B) used in the simulation. O3'(-1)-P(+1) distances of the two strands in the simulation were shown, with RMSD of the phosphodiester backbone in the inset.

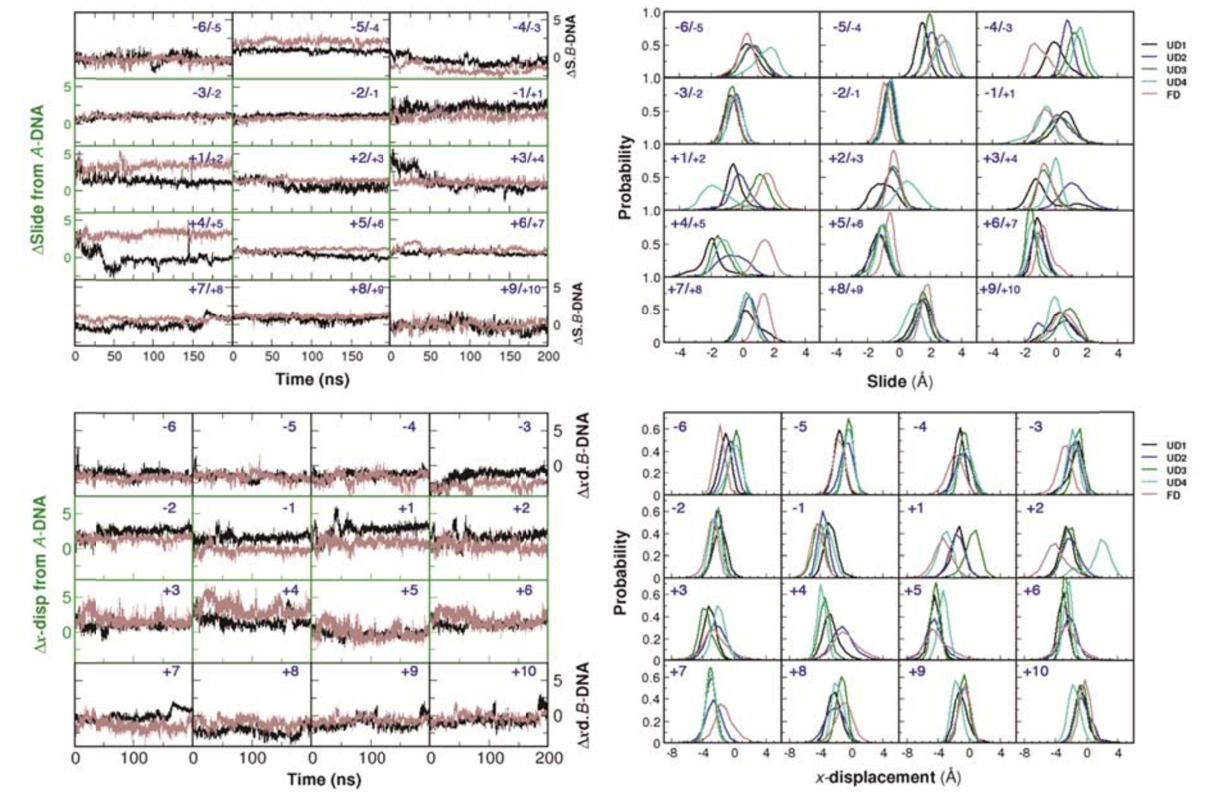

Figure S6. Early transitions in the nucleic acid conformations. *Left*, comparison of nucleic acid conformations along the 200-ns simulation of FD (brown) with the initial 200-ns of UD1 (black line), as indicated in Scheme S1. Difference in each parameter from that of the typical *A*-DNA was calculated for the +2 to +6 compartment (green frame), and difference from the value of typical *B*-DNA was calculated for the two flanking regions (black frame). *Right*, probability distributions of Slide and *x*-displacement in the 200-ns periods of simulations UD1-4 and FD.

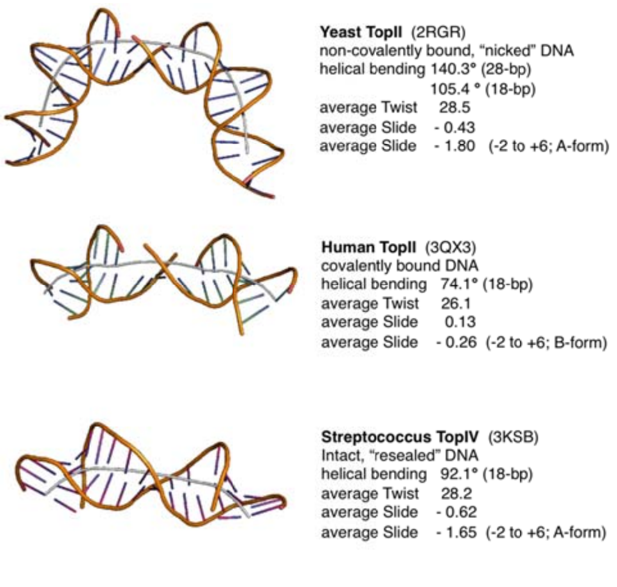

Figure S7. Crystal conformations of the gate-DNA in complex with type II topoisomerases. Helical axes were generated using Curves+.

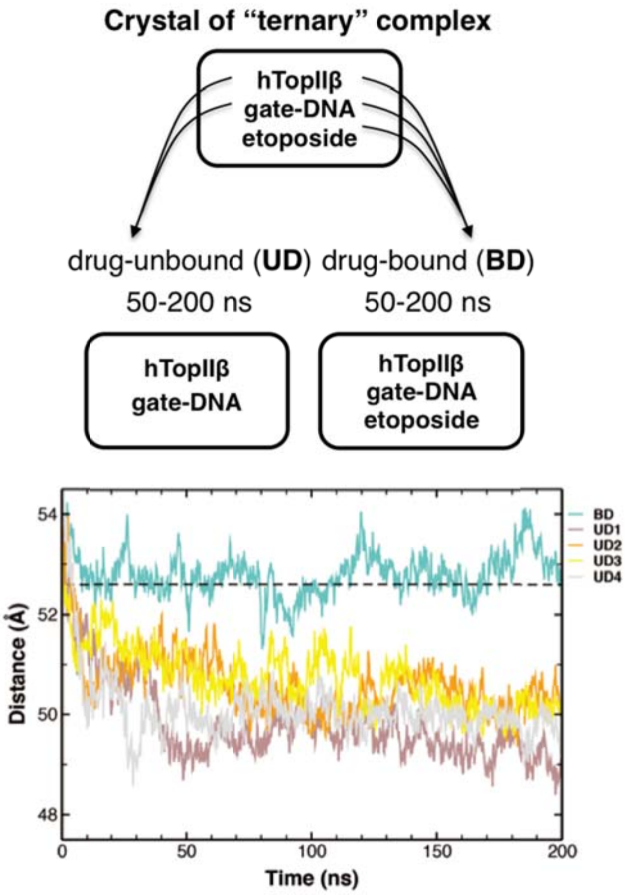

Figure S8. Inter-protomer distances of the I872 residues in the 200-ns simulations of the drug-unbound (UD1-4) and drug-bound (BD) topoisomerase IIβ•DNA intermediates demonstrate the common early transition in protein conformation. The moving average was calculated with a window size of 20.

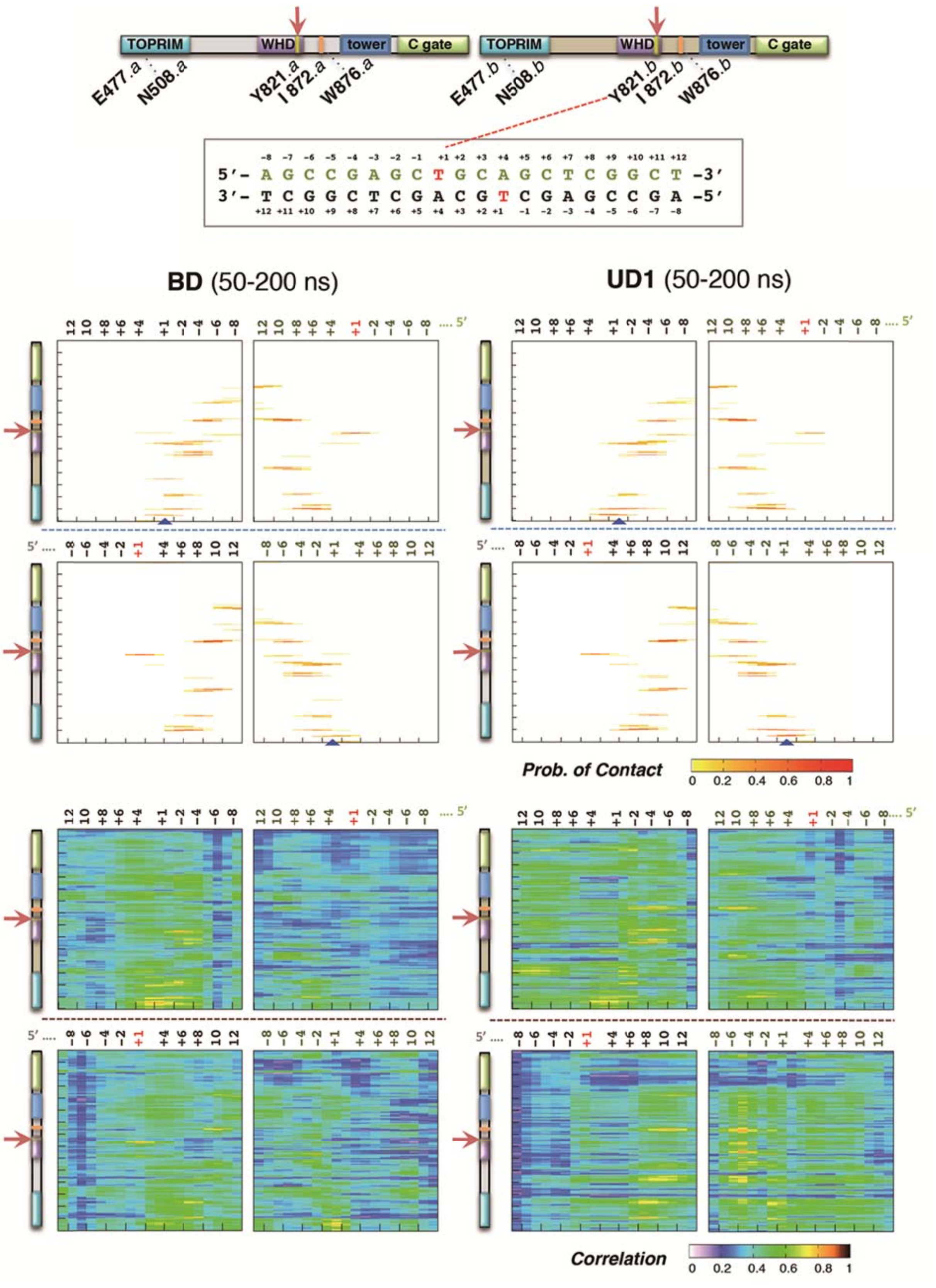

Figure S9. Probability of direct contacts (middle) and correlated motions (bottom) between topoisomerase II and the gate-DNA. The results were mapped with respect to the two polypeptide chains and the two oligonucleotide strands, giving rise to four pairing combinations for each

simulation system. The catalytic tyrosine on each peptide chain was marked with red arrow, and its bonded +1 nucleotide was highlighted in red. Blue arrowheads indicate the *in-trans* contact between E477 and the cleavage sites. The matrices of correlation coefficients are displayed in the color coding according to the instructions in *Wong B. (2010) Nature Methods 7-(8) 573*.

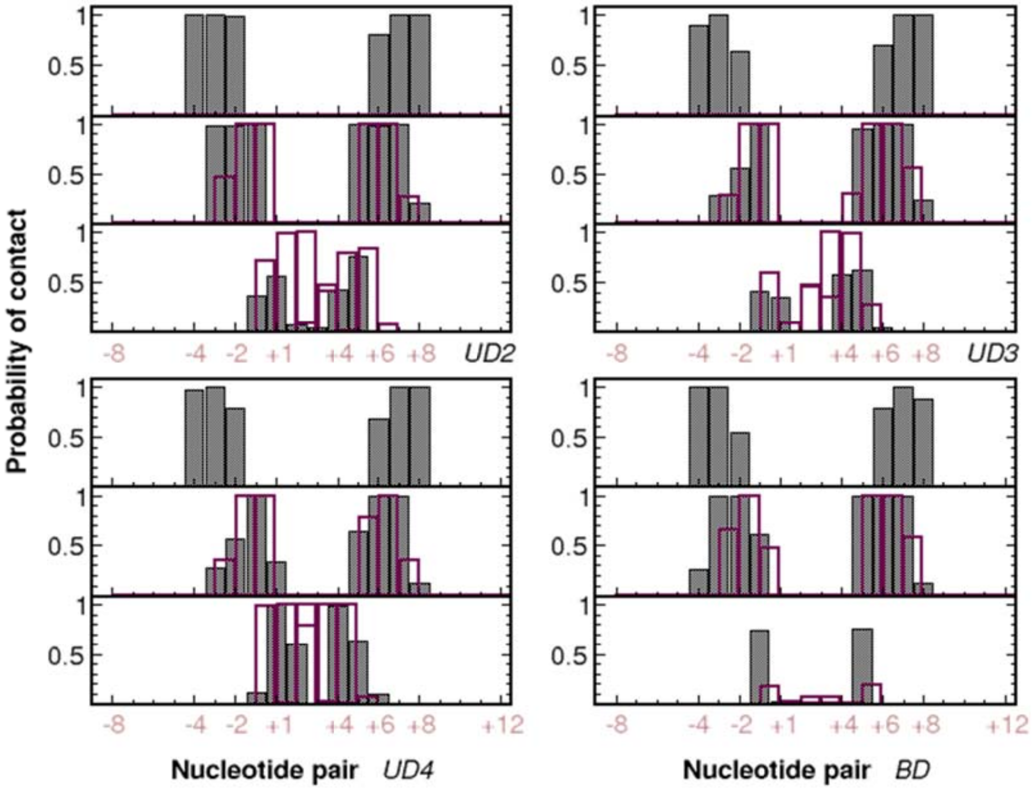

Figure S10. *In-cis* (filled) and *in-trans* (open bars) contact probabilities of Arg503 (bottom), Lys505 (middle) and Asn508 (top) with the nucleotide pairs in simulations UD2-4 and BD. Designation of the nucleotides is consistent with Fig 2 and Fig 5G.

Table S3. Probability of direct contacts between the EDxD motif (Glu477, Asp557, Asp559) and the catalytic Tyr821 with the -1 and the +1 nucleotides. Indices of the protein chains and the DNA strands are specified with the residues. The probability of contact between Tyr821 and its bonded +1 nucleotide assumes the value of 1.

#UD1

#res.chain	st1(-1)	st1(+1)	st2(-1)	st2(+1)
E477.a	0.996	0.179	0.000	0.000
D557.a	0.183	0.274	0.000	0.000
D559.a	0.394	0.000	0.000	0.000
Y821.a	0.000	0.000	0.015	1.000
E477.b	0.000	0.000	0.719	0.461
D557.b	0.000	0.000	0.023	0.030
D559.b	0.000	0.000	0.276	0.005
Y821.b	0.004	1.000	0.000	0.000

##

#UD2

#res.chain	st1(-1)	st1(+1)	st2(-1)	st2(+1)
E477.a	0.995	0.890	0.000	0.000
D557.a	0.038	0.033	0.000	0.000
D559.a	0.052	0.000	0.000	0.000
Y821.a	0.000	0.000	0.003	1.000
E477.b	0.000	0.000	0.945	0.005
D557.b	0.000	0.000	0.099	0.078
D559.b	0.000	0.000	0.376	0.003
Y821.b	0.000	1.000	0.000	0.000

##

#UD3

#res.chain	st1(-1)	st1(+1)	st2(-1)	st2(+1)
E477.a	0.991	0.001	0.000	0.000
D557.a	0.152	0.002	0.000	0.000
D559.a	0.944	0.000	0.000	0.000
Y821.a	0.000	0.000	0.006	1.000
E477.b	0.000	0.000	0.829	0.954
D557.b	0.000	0.000	0.004	0.000
D559.b	0.000	0.000	0.000	0.000
Y821.b	0.000	1.000	0.000	0.000

##

#UD4

#res.chain	st1(-1)	st1(+1)	st2(-1)	st2(+1)
E477.a	1.000	0.005	0.000	0.000
D557.a	0.333	0.004	0.000	0.000
D559.a	1.000	0.000	0.000	0.000
Y821.a	0.000	0.000	0.271	1.000
E477.b	0.000	0.000	0.616	0.937
D557.b	0.000	0.000	0.146	0.000
D559.b	0.000	0.000	0.349	0.000
Y821.b	0.000	1.000	0.000	0.000

##

#BD

#res.chain	st1(-1)	st1(+1)	st2(-1)	st2(+1)
E477.a	0.887	0.029	0.000	0.000
D557.a	0.138	0.000	0.000	0.000
D559.a	0.035	0.000	0.000	0.000
Y821.a	0.000	0.000	0.000	1.000
E477.b	0.000	0.000	1.000	0.000
D557.b	0.000	0.000	0.469	0.000
D559.b	0.000	0.000	0.077	0.000
Y821.b	0.042	1.000	0.000	0.000

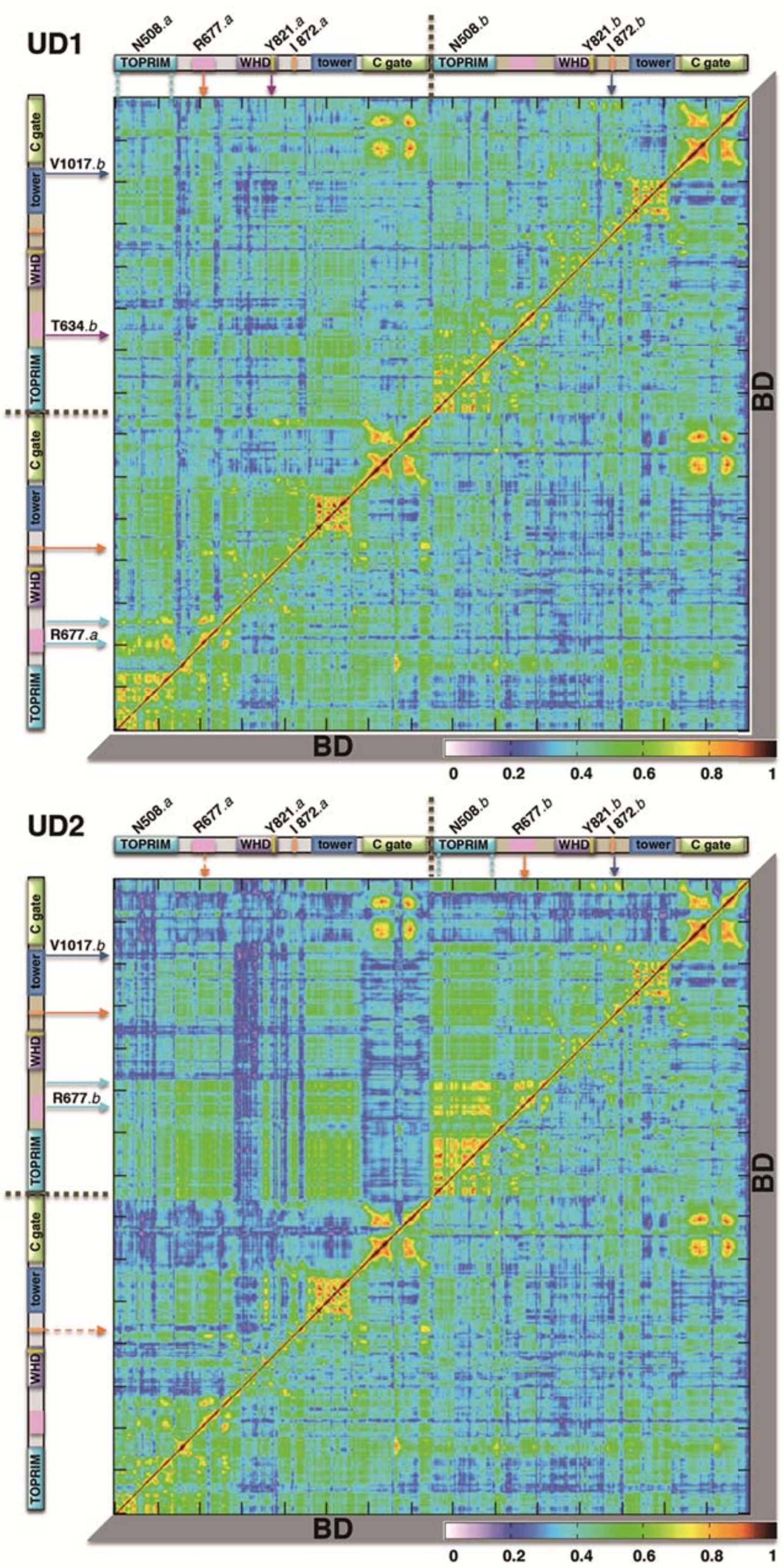

Figure S11. Correlations of the protein backbone motions in the topoisomerase II•DNA complexes. UD1 and UD2 reveal more correlated inter-domain motions of the linker (pink) in protomer A and B, respectively. The matrices of correlation coefficients are displayed in the color coding according to the instructions in *Wong B. (2010) Nature Methods 7-(8) 573*.

Table S4. Occurrence of correlated backbone motions in simulations of the drug-unbound and the drug-bound complexes.

System	UD1	UD2	UD3	UD4	BD
**Correlated protein–DNA motions** [a]	246	431	75	105	35
in-cis N^{508}—nt.(+7) (chain A / B)	1 / 1	1 / 1	0[c] / 1	1 / 1	1 / 1
in-trans N^{508}—nt.(–3) (chain A / B) [d]	0 / 0	0 / 0	0 / 0	0 / 0	0 / 0
**Linker (P^{659}…R^{677}…L^{681})-correlated** [a,b]	478	578	135	244	197
K^{505}ILN^{508}VRE^{511} (chain A / B)	82 / 2	60 / 60	4 / 19	24 / 18	4 / 14
Helix α4 (chain A / B)	256 / 52	133 / 224	6 / 83	108 / 80	79 / 100
EGI^{872}…P^{880} (chain A / B)	64 / 14	88 / 13	1 / 22	12 / 2	0 / 0
**Intercal. (EGI^{872}…P^{880})-correlated** [a,b]	2	4	0	0	0
K^{505}ILN^{508}VRE^{511} (chain A / B)	1 / 0	4 / 0	0 / 0	0 / 0	0 / 0
Helix α4 (chain A / B)	1 / 0	0 / 0	0 / 0	0 / 0	0 / 0

[a] Pairs of motions with correlation coefficients ≥ 0.7
[b] Sum of the listed inter-domain motions in chain A and chain B of the protein.
[c] Correlation coefficient = 0.64; [d] all of the *in*-trans correlation coefficients are ≤ 0.54

Animation S1. The DNA religation process revealed with the microsecond simulation of the drug-unbound complex of topoisomerase II (UD1). Crystal conformation of the drug-bound complex (green) accompanied with the modelled missing residues was used, and the two drug molecules (bright green) were removed from the complex. (*1-200 ns*) The linker (pink) between the TOPRIM (light blue) and the WHD displayed extensive correlated motions with several protein domains of the cleavage core and the DNA, with correlation linkage marked in the same color scheme as in Fig 6. The catalytic Y821 and the DNA-intercalating I872 (light orange) are shown in sphere representation. (*200-600 ns*) The conserved I506-N508 (pale green), which kept in contact with the backbone of the nucleotides flanking the conserved I872, also displayed correlated motions with these nucleotides. (*600-1000 ns*) After the middle compartment of DNA was stabilized in *A*-form, the dihedral angle comprising Cα atoms of the Gln789-Glu777•Glu777-Gln789 in the WHDs was drived toward the closed-interface configuration. The WHDs are orientated according to Fig 4D, with the crystal conformation of the helices shown in dark purple. The intercalating I872 (gray) and the catalytic Y821 (in consistent color with the helix of the same domain) are also displayed. The O3'(-1) and the P(+1) are marked as red and orange beads, respectively. (*Last snapshot)* The conformation at 1-μs of the simulation revealed the closing of the DNA gate in the drug-unbound complex of topoisomerase II by the decreased inter-subunit distances of Ile872 and those indicated in Fig 4E.
